# Different Doses of Fingolimod in Relapsing-Remitting Multiple Sclerosis: A Systematic Review and Meta-Analysis of Randomized Controlled Trials

**DOI:** 10.3389/fphar.2021.621856

**Published:** 2021-05-17

**Authors:** Xin Wu, Tao Xue, Zilan Wang, Zhouqing Chen, Xuwei Zhang, Wei Zhang, Zhong Wang

**Affiliations:** ^1^ Department of Neurosurgery and Brain and Nerve Research Laboratory, The First Affiliated Hospital of Soochow University, Suzhou, China; ^2^ Department of Neurosurgery, Suzhou Ninth People’s Hospital, Suzhou, China; ^3^ Department of Neurosurgery, Lianyungang Hospital of Traditional Chinese Medicine, Lianyungang, China

**Keywords:** fingolimod, multiple sclerosis, relapsing-remitting, different doses, systematic review

## Abstract

**Background:** The efficacy and safety of fingolimod for relapsing-remitting multiple sclerosis (RRMS) had been well verified in several large randomized controlled trials (RCTs) during the past decade. However, there are fewer systematic comparisons of different doses of fingolimod and whether the dose of 0.5 mg/d is the optimal one still remains to be solved.

**Objective:** The objective of this systematic review was to evaluate the efficacy and safety of the four existing doses of fingolimod in the treatment of RRMS, especially the dose of 0.5 mg/d.

**Methods:** MEDLINE, EMBASE, Cochrane Library, and clinicaltrials.gov were searched for RCTs which were performed to evaluate different doses of fingolimod and the corresponding control (placebo or DMTs) up to October 2020. Review Manager 5.3 software was used to assess the data. The risk ratio (RR) and mean difference (MD) was analyzed and calculated with a random effect model.

**Results:** We pooled 7184 patients from 11 RCTs. Fingolimod 0.5 mg/d was superior to control group in all eight efficacy outcomes including annualized relapse rate (ARR) (MD −0.22, 95%CI −0.29 to −0.14, *p* < 0.00001) but surprisingly showed a higher risk of basal-cell carcinoma (RR 4.40, 95%CI 1.58 to 12.24, *p* = 0.004). Although 1.25 mg/d is more than twice the dose of 0.5 mg/d, the effect size was almost similar between them. Dose of 5 mg/d obtained an unsatisfactory efficacy while showing a greater risk of adverse events than other three doses (RR 1.17, 95%CI 1.05 to 1.30, *p* = 0.003). Additionally, fingolimod 0.25 mg/d not only showed a better performance in delaying the disease progress of magnetic resonance imaging (MRI), but also achieved a certain degree of patient treatment satisfaction.

**Conclusion:** At present, 0.5 mg/d remains to be the optimal dose of fingolimod for RRMS patients but trials of a lower dose are still of great clinical significance and should be paid more attentions.

## Introduction

Multiple sclerosis (MS) is a chronic inflammatory disease that can induce the immune system to produce autoimmune responses against the central nervous system (CNS), involved both white and grey matter region, thereby slowly losing the patient's physical activity (Owens, 2016). According to a recent report by the Multiple Sclerosis International Federation (The Multiple Sclerosis International Federation, 2020), the number of MS patients globally has increased to 2.8 million in 2020, which equates to 1 in 3000 people in the world living with MS. Relapsing-remitting multiple sclerosis (RRMS) is the most common phenotype of MS. The process of onset-remission-relapse can cause progressive disability in approximately 15–30% of MS patients, thus causing great damage to the quality of life (Thompson et al., 2018). There are several disease-modyfing treatments (DMTs) but still no cure for RRMS. Therefore, researches on the drugs that can effectively control the relapse and delay the progression of RRMS are still of great clinical significance.

Fingolimod (FTY720), as the first sphingosine 1-phosphate receptor (S1PR) modulator that routinely applied in the treatment of RRMS, has been proven to be effective in reducing the patients’ annualized relapse rate (ARR) and improving the magnetic resonance imaging (MRI) performance as earlier as 2006 (Kappos et al., 2006). The prevention of lymphocyte migration out of lymphoid tissues and the directly reduction of neurodegenerative process in the CNS might be the two underlying mechanisms that allow fingolimod to exert therapeutic effect in patients with RRMS (Chun and Hartung, 2010).

During the past decade, the efficacy and the safety of fingolimod had been well verified in several large randomized controlled trials (RCTs). At the same time, many systematic reviews based on these RCTs also made a good comparison between fingolimod and other DMTs or placebo on RRMS patients (Singer et al., 2011; Roskell et al., 2012; La Mantia et al., 2016; Yang et al., 2020). However, there are fewer systematic comparisons of different doses of fingolimod. Although the dose of 0.5 mg/d is currently approved worldwide for treatment of adult RRMS, whether a lower dose of fingolimod can maintain its effectiveness while reducing adverse events and costs still remain to be revealed. Doses of fingolimod lower than 0.5 mg per day were not evaluated in the fingolimod clinical development program. We thought that a further analysis of data may be of help for dose finding, particularly to explore whether different doses may be of help for clinicians to tailor treatment to RRMS persons in case of reduced tollerability/safety and/or not optimal efficacy. Thus, we pooled data from previous RCTs and conducted a meta-analysis to investigate the differences in efficacy and safety of different doses of fingolimod.

## Methods

### Study Protocol

Before the project started, we drafted a research protocol following the Cochrane Collaboration format (Liberati et al., 2009).

### Eligibility Criteria

We set the inclusion criteria as follows: a) study type: RCT; b) language restriction: only available in English; c) participants: patients 18–65 years of age diagnosed with RRMS; d) intervention: different doses of fingolimod and the corresponding control (placebo or DMTs); e) clinical outcomes: ARR, the number of patients free of relapse and the expanded disability status scale (EDSS); MRI outcomes: number of patients free of increased gadolinium-enhanced lesions in T1, number of patients with no new or newly enlarged lesions in T2 and the percentage brain volume change (PBVC); self-report outcomes: the beck depression inventory (BDI) score and the treatment satisfaction questionnaire for medication (TSQM) score; safety outcomes: adverse events (AEs) and serious adverse events (SAEs). Included RCTs were not requested to supply all the outcomes mentioned above.

We set the exclusion criteria as follows: a) study type: retrospective studies, cohort studies, case reviews and case reports; b) participants: patients with other forms of MS; c) control: active control (i.e. that a known, effective treatment as opposed to a placebo is compared to an experimental treatment).

### Search Strategy

Two independent investigators (XW and TX) systematically searched the Clinicaltrials.gov and three main databases including MEDLINE, EMBASE, and Cochrane Library to identify relevant studies published until October 2020. The following search strategy was used (fingolimod [Title/Abstract]) AND (multiple sclerosis [Title/Abstract]) for MEDLINE; ‘fingolimod’/exp AND ‘multiple sclerosis’/exp for EMBASE; “fingolimod” in Title Abstract Keyword AND “multiple sclerosis” in Title Abstract Keyword for Cochrane Library; “fingolimod | multiple sclerosis” for Clinicaltrials.gov. Additionally, the reference lists of RCTs, relevant systematic reviews and meta-analyses were also screened independently and manually to ensure a more comprehensive search.

### Study Selection and Data Collection

According to the eligibility criteria listed above, two reviewers (XW and TX) independently evaluated all study records from the three electronic database and the reference lists of RCTs and relevant systematic reviews or meta-analyses. The duplicates and the research articles who only provided abstracts were excluded. A third reviewer (ZLW) who didn’t participate in the process of data collection would make the final decision of the disputed data when disagreements emerged among the two reviewers. After meticulous selection and evaluation, all data from the included RCTs were extracted as follows: basic information and outcome events included for each trail (Table 1), inclusion and exclusion criteria, study design, all efficacy and safety outcomes were showed in the online supplementary materials (Supplementary Table S1).

**TABLE 1:** Characteristics of the included studies and outcome events.

Study	Countries	Centers	Publications	Treatment group, (no. Of participants)	Age range	Male (%)	Mean age ±SD (year)	Study period	Outcome events
Kappos et al. (2006)	11	32	New england journal of medicine	FTY 1.25 mg (93) vs. FTY 5.0 mg (92) vs. PLA (92)	18 y-60 y	FTY 1.25 mg: 24.7	FTY 1.25 mg: 38.0 **±** 8.2	6 months	a, b, c, e, g, j
						FTY 5.0 mg: 29.3	FTY 5.0 mg: 38.3 **±** 8.5		
						PLA: 33.7	PLA: 37.1 **±** 8.7		
Cohen et al. (2010) (TRANSFORMS)	18	172	New england journal of medicine	FTY 0.5 mg (431) vs. FTY 1.25 mg (426) vs. DMT (435)	18 y-55 y	FTY 0.5 mg: 34.6	FTY 0.5 mg: 36.7 ± 8.8	12 months	a, b, c, d, e, f, j
						FTY 1.25 mg: 31.2	FTY 1.25 mg: 35.8 ± 8.4		
						DMT: 32.2	DMT: 36.0 ± 8.3		
Kappos et al. (2010) (FREEDOMS)	22	138	New england journal of medicine	FTY 0.5 mg (425) vs. FTY 1.25 mg (429) vs. PLA (418)	18 y-55 y	FTY 0.5 mg: 30.4	FTY 0.5 mg: 36.6 ± 8.8	24 months	a, c, d, e, f, j
						FTY 1.25 mg: 31.2	FTY 1.25 mg: 37.4 ± 8.9		
						PLA: 28.7	PLA: 37.2 ± 8.6		
Saida et al. (2012)	Japan	43	Multiple sclerosis journal	FTY 0.5 mg (57) vs. FTY 1.25 mg (57) vs. PLA (57)	18 y-55 y	FTY 0.5 mg: 29.8	FTY 0.5 mg: 35.0 ± 9.0	6 months	a, b, c, d, j
						FTY 1.25 mg: 31.6	FTY 1.25 mg: 36.0 ± 9.3		
						PLA: 31.6	PLA: 35.0 ± 8.9		
Calabresi et al. (2014) (FREEDOMS II)	8	117	Lancet neurology	FTY 0.5 mg (358) vs. FTY 1.25 mg (370) vs. PLA (355)	18 y-55 y	FTY 0.5 mg: 23.2	FTY 0.5 mg:40.6 ± 8.4	24 months	a, b, c, d, e, f, j
						FTY 1.25 mg: 24.1	FTY 1.25 mg: 40.9 ± 8.9		
						PLA: 18.9	PLA: 40.1 ± 8.4		
Fox et al. (2014) (EPOC)	United States and Canada	158	Multiple sclerosis and related disorders	FTY 0.5 mg (790) vs. DMT (263)	18 y-65 y	FTY 0.5 mg: 23.9	FTY 0.5 mg: 46.0 ± 9.8	6 months	g, h, i, j
						DMT: 20.9	DMT: 45.1 ± 9.8		
Comi et al. (2017) (GOLDEN)	Italy and Germany	36	Journal of neurology	FTY 0.5 mg (80) vs. DMT Filippi et al. (2014)	18 y-60 y	FTY 0.5 mg: 28.8	FTY 0.5 mg: 40.2 ± 9.1	18 months	a, b, e, f, g, j
						DMT: 32.1	DMT: 37.6 ± 9.3		
Cree et al. (2018) (PREFERMS)	United States	117	Therapeutic advances in neurological disorders	FTY 0.5 mg (436) vs. DMT (439)	18 y-65 y	FTY 0.5 mg: 28.7	FTY 0.5 mg: 41.5 ± 10.84	48 weeks	a, e, j
						DMT: 25.1	DMT: 41.9 ± 10.39		
Cree et al. (2020) (ASSESS)	6	127	JAMA neurology	FTY 0.25 mg (370) vs. FTY 0.5 mg (352) vs. DMT (342)	18 y-65 y	FTY 0.25 mg:25.4	FTY 0.25 mg: 38.9 ± 11.0	12 months	a, b, c, d, e, h, j
						FTY 0.5 mg: 25.0	FTY 0.5 mg: 40.3 ± 11.1		
						DMT: 26.3	DMT: 39.6 ± 10.8		
NCT01534182 (EPOC)	Russian	26	ClinicalTrials.gov	FTY 0.5 mg (230) vs. DMT (68)	18 y-65 y	FTY 0.5 mg: 29.6 DMT: 26.5	FTY 0.5 mg: 35.4 ± 9.9	6 months	g, h, i, j
							DMT: 36.4 ± 9.3		
NCT01317004 (EPOC)	Italy	17	ClinicalTrials.gov	FTY 0.5 mg (50) vs. DMT Liberati et al. (2009)	18 y-65 y	FTY 0.5 mg: 36.0	FTY 0.5 mg: 38.0 ± 8.7	6 months	g, h, i, j
						DMT: 27.3	DMT: 35.8 ± 7.2		

### Risk of Bias

The risk of bias plot for individual studies was assessed with the Review Manager 5.3 software. The uniform criteria to assess the risk of bias for RCTs of the Cochrane Collaboration was applied, which included: selection bias, performance bias, detection bias, attrition bias, reporting bias, and other potential biases. Each bias criterion was classified as “low”, “high”, or “unclear” after independently judged by the third reviewer.

### Summary Measures and Synthesis of Results

Review Manager 5.3 software was used to assess the data. For the dichotomous outcomes, the risk ratio ([RR]; 95% confidence interval [CI]) was analyzed and calculated with a random effect model. Mean difference (MD) was used for the continuous outcomes such as ARR, EDSS score, PBVC, BDI score and TSQM score. Heterogeneity was estimated via the I^2^ statistic, which was as follows: I^2^ < 30% suggests “low heterogeneity”; I^2^ between 30 and 50% means “moderate heterogeneity”; I^2^ > 50% denotes “substantial heterogeneity”. Sensitivity analysis was used to explore the stability of the consolidated results. For all the analyses, two tailed tests were performed and a *p* value < 0.05 was considered to be statistical significant.

## Results

**FIGURE 1:**
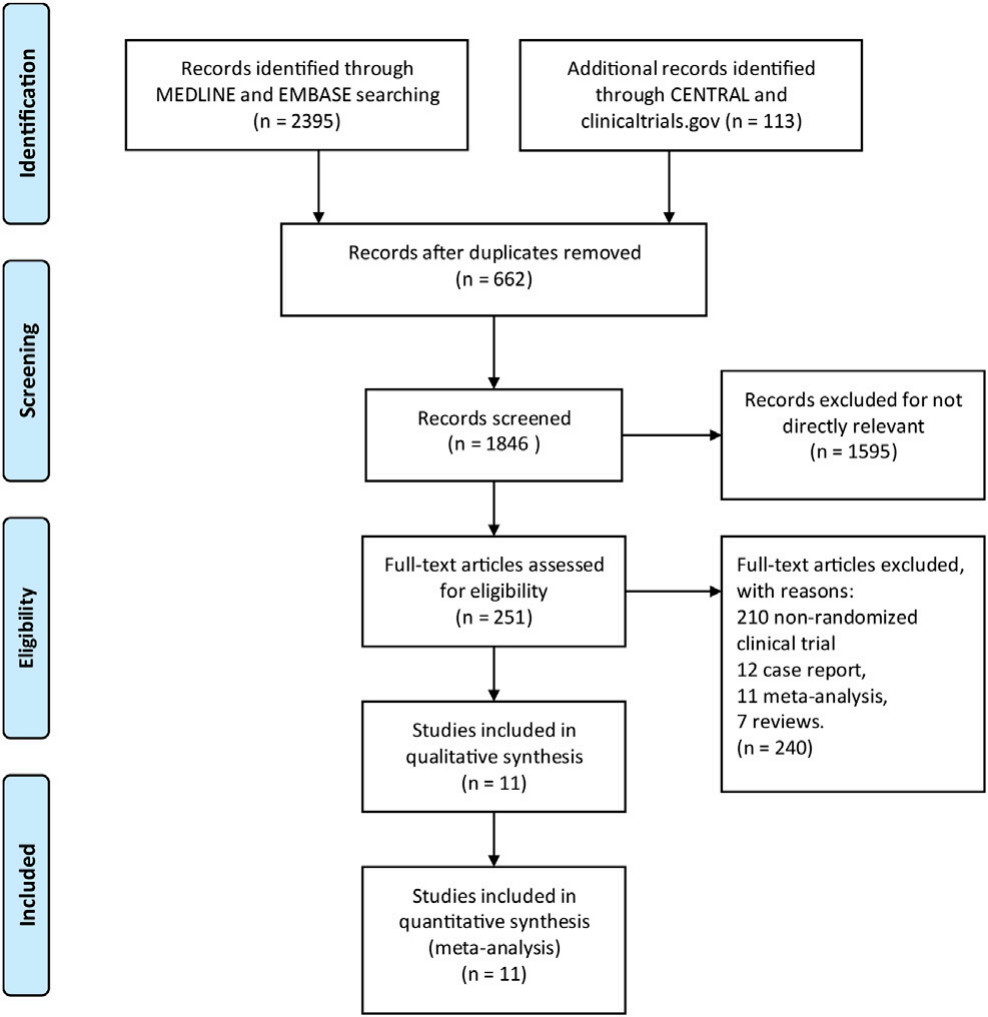
The study search, selection, and inclusion process.

A total of 2508 titles and abstracts were returned from the search through MEDLINE, EMBASE, Cochrane Library and Clinicaltrials.gov. After quick screening the titles and abstracts, a total of 2257 articles were excluded due to duplication and irrelevance and 251 full text articles were assessed for eligibility. Among them, another 240 articles were excluded due to the limitation of publication types: 210 non-randomized clinical trials, 12 case reports, 11 meta-analyses and 7 reviews. The selection process was summarized in the flow diagram (Figure 1). All 11 elected RCTs (Kappos et al., 2006), (Kappos et al., 2010), (Cohen et al., 2010; Saida et al., 2012; Calabresi et al., 2014; Fox et al., 2014; ClinicalTrials.gov, 2014; ClinicalTrials.gov, 2015; Comi et al., 2017; Cree et al., 2018; Cree et al., 2020) enrolling 7184 patients were pooled for the analyses of efficacy and safety outcomes. The main characteristics of the included 11 studies were listed in Table 1.

### Clinical Outcomes Analysis

The clinical outcomes included the ARR, the number of patients free of relapse and the EDSS score. Patients in fingolimod 0.5 mg/d and 1.25 mg/d group had significantly lower ARR and ΔEDSS (Δ means the changes from baseline to final) score than those in the control group (for the ARR: 0.5 mg/d: MD = −0.17 (−0.22 to −0.11), *p* < 0.00001; 1.25 mg/d: MD = −0.24 (−0.32 to −0.15), *p* < 0.00001; for the ΔEDSS: 0.5 mg/d: MD = −0.09 (−0.16 to −0.22), *p* = 0.01; 1.25 mg/d: MD = −0.14 (−0.21 to −0.06), *p* = 0.0003). Significant differences were also observed in the number of patients free of relapse between control group and three different doses of fingolimod group (0.5 mg/d: RR = 1.20 [1.09, 1.31], *p* = 0.0002; 1.25 mg/d: RR = 1.26 [1.14, 1.40], *p* < 0.0001; 5.0 mg/d: RR = 1.30 [1.13, 1.50], *p* = 0.0002). However, patients in fingolimod 0.25 mg/d group didn’t show significant differences in ARR and the number of patients free of relapse when compared with the patients in control group (*p* = 0.32 and 0.22, respectively). The detailed results of clinical outcomes analysis were showed in Table 2 and Supplementary Figures S1–S3.

**TABLE 2:** Effects sizes from meta-analysis of efficacy outcomes; from all trials using random effects models.

Efficacy outcomes	No. of trials contributing to the meta-analysis	No. of participants contributing to the meta-analysis	MD (95%CI)/RR [95% CI]	*p* value	I^2^ (%)
**1. ARR**					
fingolimod 0.25 mg	1	690	−0.04 (−0.12, 0.04)	0.32	N/A
fingolimod 0.5 mg	6	4060	−0.17 (−0.22, −0.11)	<0.00001	52
fingolimod 1.25 mg	3	1683	−0.24 (−0.32, −0.15)	<0.00001	53
**2. Number of patients free of relapse**					
fingolimod 0.25 mg	1	690	1.05 [0.97, 1.12]	0.22	N/A
fingolimod 0.5 mg	5	2507	1.20 [1.09, 1.31]	0.0002	69
fingolimod 1.25 mg	4	1872	1.26 [1.14, 1.40]	<0.0001	63
fingolimod 5.0 mg	1	184	1.30 [1.13, 1.50]	0.0002	N/A
**3. ΔEDSS**					
fingolimod 0.5 mg	4	2524	−0.09 (−0.16, −0.02)	0.01	0
fingolimod 1.25 mg	3	2423	−0.14 (−0.21, −0.06)	0.0003	0
**4. Number of patients free of increased gadolinium-enhanced lesions in T1**					
fingolimod 0.25 mg	1	603	1.09 [1.00, 1.19]	0.04	N/A
fingolimod 0.5 mg	5	2624	1.26 [1.14, 1.40]	<0.0001	83
fingolimod 1.25 mg	5	2150	1.42 [1.21, 1.66]	<0.0001	90
fingolimod 5.0 mg	1	158	1.74 [1.35, 2.25]	<0.0001	N/A
**5. Number of patients with no new or newly enlarged lesions in T2**					
fingolimod 0.25 mg	1	603	1.33 [1.09, 1.62]	0.005	N/A
fingolimod 0.5 mg	5	2672	1.64 [1.23, 2.19]	0.0007	88
fingolimod 1.25 mg	4	1981	1.78 [1.08, 2.96]	0.02	95
**6. PBVC**					
fingolimod 0.25 mg	1	557	−0.08 (−0.21, 0.05)	0.24	N/A
fingolimod 0.5 mg	6	3323	0.24 (0.07, 0.42)	0.006	83
fingolimod 1.25 mg	4	2176	0.37 (0.08, 0.67)	0.01	83
fingolimod 5.0 mg	1	158	−0.09 (−1.00, 0.82)	0.85	N/A
**7. ΔBDI**					
fingolimod 0.5 mg	4	1466	−1.92 (−2.77, −1.07)	<0.00001	30
**8. ΔTSQM**					
fingolimod 0.25 mg	1	381	11.10 (4.81, 17.39)	0.0005	N/A
fingolimod 0.5 mg	4	1718	13.03 (8.20, 17.85)	<0.0001	45

MD: Mean Difference; RR: Relative Risk; CI: Confidence Interval; ARR: Annualized Relapse Rate; PBVC: Percentage Brain Volume Change; EDSS: Expanded Disability Status Scale; BDI: Beck Depression Inventory; TSQM: Treatment Satisfaction Questionnaire for Medication.

### Magnetic Resonance Imaging Outcomes Analysis

The MRI outcomes included the number of patients free of increased gadolinium-enhanced lesions in T1, number of patients with no new or newly enlarged lesions in T2 and the PBVC. Compared with the control group, all fingolimod groups could both make more patients free of increased gadolinium-enhanced lesions in T1 (0.25 mg/d: RR = 1.09 [1.00, 1.19], *p* = 0.04; 0.5 mg/d: RR = 1.26 [1.14, 1.40], *p* < 0.0001; 1.25 mg/d: RR = 1.42 [1.21, 1.66], *p* < 0.0001; 5.0 mg/d: RR = 1.74 [1.35, 2.25], *p* = *p* < 0.0001), and the number of patients with no new or newly enlarged lesions in T2 was also significant smaller in those groups (0.25 mg/d: RR = 1.33 [1.09, 1.62], *p* = 0.005; 0.5 mg/d: RR = 1.64 [1.23, 2.19], *p* = 0.0007; 1.25 mg/d: RR = 1.78 [1.08, 2.96], *p* = 0.02). In addition, there was also a substantial significance in the PBVC of fingolimod 0.5 mg/d and 1.25 mg/d group when compared with the control group (0.5 mg/d: MD = 0.24 (0.07, 0.42), *p* = 0.006; 1.25 mg/d: MD = 0.37 (0.08, 0.67), *p* = 0.01). See Table 2 and Supplementary Figures S4–S6 to find the detailed results of MRI outcomes analysis.

### Patients Evaluated Outcomes Analysis

BDI and TSQM are two patients self-evaluated scales that can reflect depression and treatment satisfaction, respectively (Arnau et al., 2001), (Atkinson et al., 2004). The results showed that, compared with control group, fingolimod 0.5 mg/d could significantly reduce the depression scores (MD = −1.92 (−2.77, −1.07), *p* < 0.00001) while improving patient treatment satisfaction (MD = 13.03 (8.20, 17.85), *p* < 0.0001). In the meanwhile, patients in fingolimod 0.25 mg/d group were also more willing to give a higher score in the TSQM scale (MD = 11.10 (4.81, 17.39), *p* = 0.0005). The detailed results of patients evaluated outcomes analysis were also showed in Table 2 and Supplementary Figures S7,S8.

### Safety Outcomes

The safety outcomes were assessed by adverse events and serious adverse events. We combined the data collected from the 11 trials and found that fingolimod 5.0 mg/d showed a significant high risk of the adverse events (RR = 1.17 [1.05, 1.30], *p* = 0.003, Table 3 and Supplementary Figure S9). In addition, for the serious adverse events, fingolimod 0.5 mg/d also showed a significant high risk compared with the control group (RR = 1.25 [1.01, 1.54], *p* = 0.04, Table 3 and Supplementary Figure S10). Thus, we further sub-analyzed the specific serious adverse event that at least two trials provided data in the fingolimod 0.5 mg/d group. The results showed that compared with placebo or DMTs group, the patients in fingolimod 0.5 mg/d group were more likely to have basal-cell carcinoma, which made major contribution to the high risk of serious adverse events (RR = 4.40 [1.58, 12.24], *p* = 0.004, Table 4 and Supplementary Figures S11–S14).

**TABLE 3:** Effects sizes from meta-analysis of safety outcomes; from all trials using random effects models.

Safety outcomes	No. of trials contributing to the meta-analysis	No. of participants contributing to the meta-analysis	RR [95% CI]	*p* value	I^2^ (%)
**1. AEs**					
fingolimod 0.25 mg	1	690	1.01 [0.96, 1.07]	0.72	N/A
fingolimod 0.5 mg	10	5594	1.05 [0.99, 1.11]	0.09	85
fingolimod 1.25 mg	5	2721	1.01 [0.98, 1.04]	0.46	39
fingolimod 5.0 mg	1	187	1.17 [1.05, 1.30]	0.003	N/A
**2. SAEs**					
fingolimod 0.25 mg	1	690	1.42 [0.83, 2.43]	0.20	N/A
fingolimod 0.5 mg	10	5594	1.25 [1.01, 1.54]	0.04	0
fingolimod 1.25 mg	5	2721	1.38 [0.96, 1.98]	0.09	44
fingolimod 5.0 mg	1	187	2.14 [0.85, 5.40]	0.11	N/A

RR: Relative Risk; CI: Confidence Interval; AEs: Adverse Events; SAEs: Serious Adverse Events.

**TABLE 4:** Meta-analysis of SAEs between fingolimod 0.5 mg/d and placebo or DMTs groups; from all trials using random effects models, where at least two trials provided data that could be included.

SAE	No. of trials contributing to the meta-analysis	No. of participants contributing to the meta-analysis	RR [95% CI]	*p* value	I^2^ (%)
Infection	6	4007	1.29 [0.41, 4.02]	0.66	37
MS relapse	6	4175	0.65 [0.35, 1.21]	0.17	0
Bradycardia	4	2530	2.97 [0.75, 11.72]	0.12	0
Death	4	3444	0.20 [0.01, 4.09]	0.29	N/A
Basal-cell carcinoma	4	3085	4.40 [1.58, 12.24]	0.004	0
Atrioventricular block	3	1687	2.03 [0.45, 9.25]	0.36	0
Chest pain	3	2584	2.25 [0.60, 8.49]	0.23	0
Lymphopenia	3	1932	1.35 [0.16, 11.31]	0.78	0
Epilepsy	3	2225	2.92 [0.46, 18.48]	0.25	0
Depression	3	2417	0.67 [0.08, 6.03]	0.72	48
Dyspnea	2	1529	4.70 [0.23, 97.46]	0.32	N/A
Breast cancer	2	1703	0.83 [0.02, 27.60]	0.92	64
Melanoma	2	1703	0.33 [0.01, 8.03]	0.49	N/A
Abortion	2	1556	0.30 [0.05, 1.94]	0.21	0
Syncope	2	1382	0.74 [0.07, 7.34]	0.79	31
Macular edema	2	1556	0.99 [0.06, 15.79]	1.00	N/A

SAE: Serious Adverse Event; RR: Relative Risk; CI: Confidence Interval; MS: multiple sclerosis; N/A: Not Applicable.

### Risk of Bias in Included Studies

**FIGURE 2:**
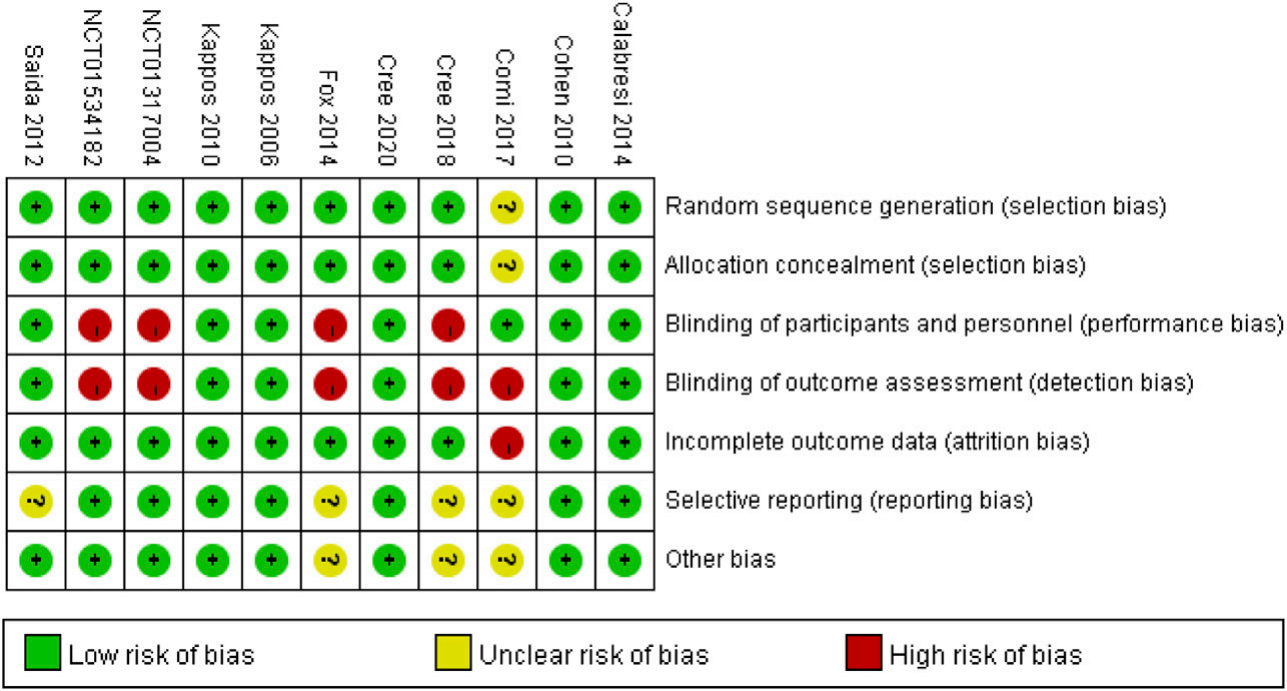
Risk of bias: A summary table for each risk of bias item for each study.

Full details of the risk bias for all enrolled studies were showed in Figure 2. One clinical trials showed unclear risk of bias both in random sequence generation and allocation concealment. For the blinding of participants and personnel, the risk of bias was high in five studies. For the blinding of outcome assessment, the risk of bias was high in four trials. For the incomplete outcome data, the risk of bias was low in all eleven trials. For selective reporting, the risk of bias was unclear in four study. Apart from these items, unclear risk of bias was also observed in three RCTs.

## Discussion

Nowadays, oral fingolimod is widely used in the treatment of RRMS and has achieved good therapeutic effects. Several large clinical trials have shown that fingolimod with once-daily doses of 0.5, 1.25 and 5 mg could both significantly lower the ARR compared with the placebo group (Kappos et al., 2006), (Kappos et al., 2010). Moreover, in response to the recommendation of US Food and Drug Administration, study that evaluated fingolimod with once-daily dose of 0.25 mg has recently appeared (Cree et al., 2020). For a more comprehensive comparison in the efficacy of different doses of fingolimod, a total of eight outcomes were included in this meta-analysis and were divided into three major parts: clinical outcomes, MRI outcomes and self-report outcomes. We conducted this meta-analysis for the purpose of evaluating the efficacy and safety of the four existing doses. On the basis of our data pooled from the eleven published RCTs, we found fingolimod with once-daily dose of 0.5 mg was superior to placebo or DMTs in all efficacy terms that included in this study but showed a higher risk of serious adverse events. At the same time, although 1.25 mg is more than twice the dose of 0.5 mg, the effect size in the reduction of clinical relapse and the amelioration of MRI performance was almost similar between them. As the largest dosage, fingolimod 5 mg/d certainly showed the highest MD in the number of patients free of clinical relapse and increased gadolinium-enhanced lesions in T1 compared with placebo or DMTs. Nevertheless, this dose also means a greater financial burden for the patients or public health system, and a higher risk of adverse events than other doses according to our analysis. In comparison with placebo or DMTs, fingolimod 0.25 mg/d not only showed a better performance in MRI parameters, but also achieved a certain degree of patient treatment satisfaction. Therefore, it is necessary to conduct more studies in the future to evaluate the efficacy and safety of fingolimod 0.25 mg/d.

As the most commonly reported clinical outcome in RRMS trials, ARR was widely selected in systematic reviews (Rae-Grant et al., 2018), (Fogarty et al., 2016). Combined with the number of patients free of relapse, investigators could intuitively evaluate the relapse level of patients. In this study, patients treated with fingolimod 0.5 mg/d and 1.25 mg/d showed a significant lower relapse level compared with placebo or DMTs. In addition, the inclusion criteria of almost all RRMS trials both included the EDSS scores, which ranged from 0 to 10 and was positively related to the degree of disability (Kurtzke, 1983). An increase greater or equal to one point in the EDSS score after three months was considered as disability progression if the most inclusive definition was acceptable (Lorscheider et al., 2016). The MD in ΔEDSS of fingolimod 0.5 mg/d and 1.25 mg/d were both negative in this study, which indicated that fingolimod 0.5 and 1.25 mg/d could effectively prevent or retard the progression of disability when compared with placebo or DMTs.

For MRI outcomes, we included number of patients free of increased gadolinium-enhanced lesions in T1, number of patients with no new or newly enlarged lesions in T2 and the PBVC. Gadolinium-enhanced T1 lesions and new or newly enlarged T2 lesions in MRI are considered as a measure of the focal inflammatory activity (Simon, 2014). Because of the higher sensitivity than clinical assessment in detecting MS disease activity, they sometimes can surrogate ARR and serve as the primary outcomes in RRMS trials (Filippi et al., 2014). All doses even 0.25 mg/d of fingolimod both performed better than placebo or DMTs, as fingolimod induced more patients free of MS disease activity in T1 and T2. Brain volume change is another recognized MRI marker that can reflect both focal and diffuse pathology in MS(De Stefano et al., 2014), (Filippi et al., 2019). A post hoc analysis of FREEDOMS trail (Kappos et al., 2010) found that brain volume loss was associated to the disability progression and could further worsen disability over a longer period of time (Jeffery et al., 2016). Among the four doses of fingolimod, only 0.5 mg/d and 1.25 mg/d had a significant decreased brain volume loss than the placebo or DMTs group. Thereby, although the dose of 0.25 mg/d might be able to maintain the stability of the disease for a certain duration, its effectiveness in long-term brain volume loss and accompanying disability progression was still worse than 0.5 and 1.25 mg/d.

As for the patients evaluated outcomes, TSQM score and BDI score were selected for analysis. TSQM score was both evaluated as primary study subject in the three included EPOC trails that were separately conducted in USA, Russian and Italy (Fox et al., 2014; ClinicalTrials.gov, 2014; ClinicalTrials.gov, 2015). A higher TSQM score indicated a greater treatment satisfaction. Patients treated with fingolimod 0.25 and 0.5 mg/d showed a significant greater extent of improvement in ΔTSQM than the patients treated with placebo or DMTs. The better effectiveness as we mentioned earlier, the fewer side effects and a more convenient way of administration may be the reasons why fingolimod was superior to other injectable DMTs in terms of patient satisfaction. Depression is common psychiatric comorbidities in patients with MS and MS-related immune-inflammatory, and brain structural alterations might play a role of its pathogenesis (Solaro et al., 2018), (Rossi et al., 2017). The incidence of depression would increase shortly after or during the disease exacerbations in RRMS(Moore et al., 2012). The current study used the BDI score to investigate the efficacy of fingolimod 0.5 mg/d in MS-related depression compared with placebo or DMTs. With a MD of −1.92, fingolimod 0.5 mg/d demonstrated a numerically larger scores reduction from baseline to the final.

Regarding safety, fingolimod 0.5 and 5 mg/d were found a significant higher risk of SAEs and AEs when compared with placebo or DMTs, respectively. After sub-analyzing the specific SAE in fingolimod 0.5 mg/d group, we found that the risk of basal-cell carcinoma was much higher than other SAEs. As with many immunodulatory agent, the effect of fingolimod on the immune system might confer a greater risk of malignancy including the basal-cell carcinoma (Cohen et al., 2019). Furthermore, the current findings were also consistent with a long-term extension study that the basal-cell carcinoma was the most common SAEs, in which all patients were treated with oral fingolimod 0.5 mg once daily (Cohen et al., 2019). Despite this, 0.5 mg/d still seems to be the best dose choice of fingolimod for RRMS patients at present.

Several limitations of the present meta-analysis should not be ignored. Firstly, the analysis of the dose of 0.25 and 5 mg/d were performed based on limited data. Only one published RCT with 370 and 92 patients were pooled to test the efficacy and safety of 0.25 and 5 mg/d, respectively. Secondly, this meta-analysis was not registered prior to the data collection. Thirdly, high level of heterogeneity was found in several data. As we showed in Table 1 and Supplementary Table S1, the variation in the study designs, the inclusion and exclusion criteria, the baseline characteristics of mean age and the region of study, and especially the duration of the trials might make the explanation. For the data with heterogeneity greater than 50%, sensitivity analysis was performed. After we excluded the trial by Cohen et al.^12^, the heterogeneity of the ARR in the 1.25 mg/d group changed from 64 to 53% (Supplementary Figure S15). Other sensitivity analyses demonstrated that all the statistics were robust (Supplementary Figures S16,S17).

## Conclusion

In conclusion, the present study indicated that combined safety and the efficacy comprehensively obtained from clinical outcomes, MRI outcomes and patients evaluated outcomes, 0.5 mg/d remains to be the optimal dose of fingolimod for RRMS patients so far. Besides, trials of the dose lower than 0.5 mg/d are still of great clinical significance and should be paid more attentions.

## Data Availability

The original contributions presented in the study are included in the article/Supplementary Material, further inquiries can be directed to the corresponding authors.
