# Supplementary material for: Different Doses of Fingolimod in Relapsing-Remitting Multiple Sclerosis: A Systematic Review and Meta-Analysis of Randomized Controlled Trials
Source: Front Pharmacol. 2021 May 17;12:621856. doi: 10.3389/fphar.2021.621856 (PMC8165387; doi:10.3389/fphar.2021.621856)
Supplement: Supplementary file 1 [file DataSheet1.PDF]

Table S1: Inclusion, exclusion criteria, study design and outcome assessments of the included studies

**Trails**	**Kappos et al 2006**
Inclusion Criteria	Patients aged 18y to 60y with RRMS and at least one of the following: two or more documented relapses during the previous 2 years, one or more documented relapses in the year before enrollment, and one or more gadolinium-enhanced lesions detected on MRI at screening. Additional eligibility criteria included a score of 0 to 6 on the EDSS and with no evidence of relapse for at least 30 days before screening and during the screening and baseline phases.
Exclusion Criteria	Previously use of corticosteroids (within 30 days), immunomodulatory therapy (within 3 months), or immunosuppressive treatment (e.g., azathioprine or methotrexate within 6 months, cyclophosphamide within 12 months, or mitoxantrone or cladribine within 24 months); a history of cardiac conditions that might increase the risk of a decrease in heart rate; a white-cell count of less than 3500 per cubic millimeter; and a lymphocyte count of less than 800 per cubic millimeter.
Study Design	Patients were randomly assigned, in a 1:1:1 ratio, to 1.25 mg of fingolimod, 5.0 mg of fingolimod, or a matching placebo once daily; all drugs were given as capsules.
Efficacy Outcomes	The total number of gadolinium-enhanced lesions per patient recorded on T1-weighted MRI at monthly intervals for 6 months; the total volume of gadolinium-enhanced lesions per patient; the proportion of patients with gadolinium-enhanced lesions; the total number of new lesions per patient on T2-weighted images; changes in lesion volume on T2-weighted images; the brain volume from baseline to month 6; the number of patients remaining free of relapse; the ARR; the time to the first relapse; the Hamburg Quality of Life Questionnaire in Multiple Sclerosis score and the BDI score.
Safety Outcomes	Adverse events and serious adverse events.

**Trails**	**Cohen et al 2010 (TRANSFORMS)**
Inclusion Criteria	Patients aged 18y to 55y with RRMS and had at least one documented relapse during the previous year or at least two documented relapses during the previous 2 years, and had a score of 0 to 5.5 on the EDSS.
Exclusion Criteria	A documented relapse or corticosteroid treatment within 30 days before randomization, active infection, macular edema, immunosuppression (either drug- or disease-induced), and clinically significant coexisting systemic disease.
Study Design	Patients were randomly assigned to 12 months of treatment with oral fingolimod, at a daily dose of either 1.25 or 0.5 mg, or intramuscular interferon beta-1a, at a weekly dose of 30 μg.
Efficacy Outcomes	The ARR; the number of new or enlarged hyperintense lesions on T2-weighted MRI scans at 12 months and the time to confirmed disability progression.
Safety Outcomes	Adverse events and death.

**Trails**	**Kappos et al 2010 (FREEDOMS)**
Inclusion Criteria	Patients aged 18y to 55y with RRMS and had one or more documented relapses in the previous year or two or more in the previous 2 years; and a score of 0 to 5.5 on the EDSS.
Exclusion Criteria	Relapse or corticosteroid treatment within 30 days before randomization, active infection, macular edema, diabetes mellitus, immune suppression (drug- or disease-induced), or clinically significant systemic disease.
Study Design	Patients were randomly assigned, in a 1:1:1 ratio, to receive oral fingolimod capsules in a dose of 0.5 mg or 1.25 mg or matching placebo, once daily for 24 months.
Efficacy Outcomes	The ARR; the time to confirmed disability progression; the time to a first relapse; time to disability progression (confirmed after 6 months); changes in the EDSS score and MSFC z score between baseline and 24 months; number of gadolinium-enhancing lesions; proportion of patients free from gadolinium-enhancing lesions; number of new or enlarged lesions on T2-weighted MRI scans; proportion of patients free from new or enlarged lesions on T2-weighted scans; volumes of hyperintense lesions on T2-weighted scans and hypointense lesions on T1-weighted scans; change in brain volume between baseline and 24 months.
Safety Outcomes	Adverse events and death.

**Trails**	**Saida et al 2012**
Inclusion Criteria	Patients aged 18y to 60y with RRMS and had one or more documented relapses in the previous year or two or more in the previous 2 years, or at least one gadolinium (Gd)-enhanced T1-weighted brain lesion within the 30 days prior to study commencement; had to have at least one T2-weighted brain lesion and a score of 0–6 on the EDSS.
Exclusion Criteria	Patients with long cord lesions of at least three vertebral segments on spinal MRI at or prior to screening; primary progressive MS, relapse or corticosteroid treatment within 30 days before randomization, malignancy, macular oedema, diabetes mellitus, active infection, immune suppression (drug or disease induced), clinically significant systemic disease, or pregnancy; received cladribine, cyclophosphamide, mitoxantrone, or other immunosuppressive or immunoglobulin medication in the six months prior to randomization; had plasmapheresis immunoadsorption or interferon beta therapy in the three months prior to randomization.
Study Design	Patients were randomly assigned, in a 1:1:1 ratio, to receive once-daily fingolimod capsules, 0.5 mg or 1.25 mg, or matching placebo for six months.
Efficacy Outcomes	The percentage of patients free from Gd-enhanced lesions at both months 3 and 6; the percentage of patients free from relapses over six months; the number of Gd-enhanced lesions at six months; the number of new/enlarging T2 lesions over six months; the proportions of patients free from new/enlarging T2 lesions over six months; the proportions of patients free from new MRI activity (Gd-enhanced lesions and new/enlarging T2 lesions); the ARR.
Safety Outcomes	Adverse events and serious adverse events.

Trails	Calabresi et al 2014<br>(FREEDOMS II)
Inclusion Criteria	Patients aged 18y to 55y with RRMS and had one or more confirmed relapses during the preceding year (or two or more confirmed relapses during the previous 2 years), had an EDSS 10 score of 0–5.5, and had no relapse or steroid treatment within 30 days before randomization.
Exclusion Criteria	Patients with clinically significant systemic disease or immune suppression (drug-induced or disease induced), active infection or macular oedema, diabetes mellitus, or a history of malignancy (apart from<br>successfully treated basal or squamous-cell skin carcinoma), and patients with specific cardiac, pulmonary, or hepatic disorders.
Study Design	A 24-month, randomized, double-blind, placebo-controlled, parallel-group, multicentre study comparing the efficacy of once-daily fingolimod (0.5 mg and 1.25 mg doses) with placebo.
Efficacy Outcomes	The ARR; percent brain-volume change from baseline and time to disability progression; the time to first relapse and proportion of relapse free patients; time to disability progression confirmed at 6 months, as measured by EDSS; change from baseline to the end of study on the MSFC score; and effect on MRI measurements of inflammatory disease activity, and MRI measurements of burden of disease.
Safety Outcomes	Adverse events and death.

Trails	Fox et al 2014<br>(EPOC)
Inclusion Criteria	Patients had been treated with an DMT for at least 6 months before screening and entered the study without an intervening washout period; adults under 65 years of age with RRMS; an EDSS score of 0–5.5, and treatment-naïve to fingolimod.
Exclusion Criteria	History of chronic disease of the immune system (except MS), history of active malignancy (except localized basal/ squamous cell carcinoma of the skin), uncontrolled diabetes mellitus (glycosylated hemoglobin > 7%), macular edema present at screening, active systemic bacterial, viral, or fungal infection (s), pregnancy, and certain cardiovascular, pulmonary, and hepatic conditions.
Study Design	A 6-month, randomized, open-label, multicenter trial with an optional 3-month extension, patients were randomized using an interactive voice response system to either the once-daily fingolimod 0.5mg arm or the DMT arm,in a 3:1 ratio.
Efficacy Outcomes	Changes in treatment satisfaction between baseline and month 6 measured by TSQM; changes in fatigue severity scale, depression (BDI), activities of daily living, and health-related QOL (SF-36) between baseline and month 6; the seven-point clinical global impression of improvement scale.
Safety Outcomes	Adverse events and death.

**Trails**	**Comi et al 2017 (GOLDEN)**
Inclusion Criteria	Patients aged 18 to 60 years and diagnosed with RRMS with active disease (at least one clinical relapse in the past year, or two clinical relapses in the past 2 years if there were signs of disease activity in one brain MRI scan performed in the past 6 months) and cognitive impairment at screening.
Exclusion Criteria	Unsatisfactory response with multi-weekly IFNs (IFN β-1a/b); hyperactive forms of MS; EDSS score > 5.0; acute MS relapse < 30 days before screening; prior or current diagnosis of major depression according to the Diagnostic and Statistical Manual of Mental Disorders-Text Revision and history of any chronic disease of the immune system other than MS.
Study Design	An 18-month multicentre, open-label, rater-blinded, randomised, parallel-group pilot study conducted in patients with RRMS.
Efficacy Outcomes	Cognitive impairment; executive functions; depression score; MRI parameters (T2-hyperintense, T1-hypointense and T1-enhancing lesions, normalised brain volume and percentage brain volume change versus screening scan); MS relapses; EDSS scores.
Safety Outcomes	Adverse events and serious adverse events.

**Trails**	**Cree et al 2018 (PREFERMS)**
Inclusion Criteria	Patients aged 18 – 65 years, diagnosed with RRMS and with an EDSS score up to 6; Patients naïve to treatment or who have been treated with no more than one class of DMT previously.
Exclusion Criteria	Use of other investigational drugs within 30 days of screening; history of hypersensitivity to any of the study drugs or to drugs of similar chemical classes; prior exposure to fingolimod or any other sphingosine-1-phosphate-receptor-modulating compounds; history or presence of malignancy of any organ system; patients diagnosed with secondary progressive MS or primary progressive MS; patients with a history of chronic disease of the immune system other than MS or a known immunodeficiency syndrome; patients with uncontrolled diabetes mellitus (glycated hemoglobin >7%); diagnosis of macular edema during the screening phase; patients with active systemic bacterial, viral, or fungal infections; patients without a history of chickenpox or without vaccination against varicella-zoster virus at screening; patients who have received any live or live attenuated vaccines; patients with any medically unstable condition as assessed by the investigator.
Study Design	A randomized, open-label, active-controlled, parallel-group, multicenter study that followed up patients for 48 weeks, patients were randomized 1:1 to fingolimod 0.5mg/day or to an DMT using an interactive voice-and-web-response system.
Efficacy Outcomes	Patient retention on randomized treatment over 48 weeks; reasons for discontinuation of randomized treatment; cognitive impairment (SDMT); changes in brain volume (measured as percentage change from baseline using MRI); and patient-reported satisfaction; the ARR; cumulative number of newly active gadolinium-enhanced (Gd+) T1 lesions; number of new/enlarged T2 lesions; number of new active lesions; change in Gd+ lesion count; and changes in cortical graymatter and thalamic volume measures.
Safety Outcomes	Adverse events and serious adverse events.

**Trails**	**Cree et al 2020 (ASSESS)**
Inclusion Criteria	Eligible participants were between ages 18 and 65 years, had received a diagnosis of RRMS, had experienced at least 1 documented relapse during the previous year or 2 documented relapses during the previous 2 years before randomization, had received an EDSS score between 0 and 6.0 points (both inclusive) at screening, and had not experienced a relapse within 30 days of randomization.
Exclusion Criteria	Patients with a history of malignancy other than cutaneous basal cell carcinoma in the last 5 years, active infection, diabetes mellitus, macular edema or clinically significant systemic disease, active chronic diseases of the immune system other than MS, severe hepatic injury or other hepatic conditions, certain electrocardiogram findings, or history of certain cardiovascular conditions within 6 months were excluded. Patients were also excluded if they received the following treatments prior to randomization: immunosuppressive/chemotherapeutic medications within 6 months; immunoglobulins within 4 weeks; natalizumab within 2 months; rituximab, alemtuzumab, ofatumumab, ocrelizumab, or cladribine within one year, or teriflunomide within 3.5 months.
Study Design	A phase 3b multicenter randomized parallel-group rater-blinded and dose-blinded 12-month clinical trial, eligible adult participants were randomized (1:1:1) to receive fingolimod, 0.5mg, or fingolimod, 0.25 mg, orally once per day or glatiramer acetate, 20 mg, subcutaneously once per day using an interactive voice response system.
Efficacy Outcomes	The ARR; the number of new or newly enlargingT2 lesions, the number of gadolinium-enhancing T1 lesions and the proportion of participants free from these lesions, the volume of gadolinium-enhancing T1 lesions, the change from baseline in T2 lesion volume, the T1-hypointense lesion volume and brain volume, the change from baseline in the TSQM; scores from the MSFC; the SDMT score; the Patient-Reported Indices in Multiple Sclerosis-Activities scale; the 29-item Multiple Sclerosis Impact Scale; the proportion of relapse-free participants, the time to first relapse, and the severity of relapses.
Safety Outcomes	Adverse events and serious adverse events.

**Trails**	**NCT01534182**<br>**(EPOC)**
Inclusion Criteria	Patients must be diagnosed with RRMS;<br>Patients who explicitly agree to be assigned to a treatment group that may receive or DMT after having been informed about their respective benefits and possible adverse events by the investigator;<br>Male or female patients aged 18-70 years;<br>An EDSS score of 0-6 inclusive;<br>Must have received continuous treatment with a single approved and indicated MS DMT for a minimum of 6 months prior to the screening visit;<br>Patients must continue with this MS DMT until the randomization visit;<br>Naïve to treatment with fingolimod.
Exclusion Criteria	A manifestation of MS other than those defined in the inclusion criteria; A history of chronic disease of the immune system other than MS or a known immunodeficiency syndrome; History of malignancy of any organ system; Diagnosis of macular edema during Screening Phase; Patients with active systemic bacterial, viral or fungal infections, or known to have AIDS or to have positive HIV antibody test; Patients who have received any live or live attenuated vaccines (including for varicella-zoster virus or measles) within 2 months prior to baseline; Patients who have received total lymphoid irradiation or bone marrow transplantation; History of selected immune system treatments and/or medications; Any medically unstable condition, as assessed by the investigator; Selected cardiovascular, or hepatic conditions; Selected abnormal laboratory values; Patients with any other disease or clinical condition (including neurologic or psychiatric disorders) which may affect patient enrollment into the study and study medication use by the Investigators' opinion; Participation in any clinical research study evaluating another not approved in Russia investigational drug or therapy within 6 months prior to baseline; History of hypersensitivity to the study drug or to drugs of similar chemical classes; Pregnant or nursing (lactating) women.
Study Design	A 6-month, randomized, active comparator, open-label, multi-center study to evaluate patient outcomes, safety and tolerability of fingolimod 0.5 mg/day in patients with relapsing remitting multiple sclerosis who are candidates for multiple sclerosis therapy change from previous DMT.
Efficacy Outcomes	Change in patient-reported treatment satisfaction (TSQM); changes in patient-reported effectiveness, side effects and convenience; change in patient-reported depression (BDI); change in patient-reported health-related quality-of-life using the SF-36.
Safety Outcomes	Adverse events, serious adverse events and death.

Trails	NCT01317004<br>(EPOC)
Inclusion Criteria	Patients must be diagnosed with RRMS;<br>Patients who explicitly agree to be assigned to a treatment group that may receive or DMT after having been informed about their respective benefits and possible adverse events by the investigator;<br>Male or female patients aged 18-65 years;<br>An EDSS score of 0-5.5 inclusive;<br>Must have received continuous treatment with a single approved and indicated MS DMT for a minimum of 6 months prior to the screening visit;<br>Patients must continue with this MS DMT until the randomization visit;<br>Naïve to treatment with fingolimod.
Exclusion Criteria	A manifestation of MS other than those defined in the inclusion criteria;<br>A history of chronic disease of the immune system other than MS or a known immunodeficiency syndrome;<br>History of malignancy of any organ system (other than localized basal cell carcinoma of the skin), treated or untreated, within the past 5 years, regardless of whether there is evidence of local recurrence or metastases;<br>Patients with uncontrolled diabetes mellitus (HbA1c > 7%);<br>Diagnosis of macular edema during Screening Phase.
Study Design	A 6-month, randomized, active comparator, open-label, multi-center study to evaluate patient outcomes, safety and tolerability of fingolimod (FTY720) 0.5 mg/day in patients with relapsing remitting multiple sclerosis who are candidates for MS therapy change from previous DMT.
Efficacy Outcomes	Change in patient-reported treatment satisfaction (TSQM); change from baseline in patient-reported activities of daily living; change from baseline in patient-reported fatigue; changes in patient-reported effectiveness, side effects and convenience; change in patient-reported depression (BDI); change in patient-reported health-related quality-of-life using the SF-36; physician-reported clinical global impression of improvement.
Safety Outcomes	Adverse events, serious adverse events and death.

EDSS: Expanded Disability Status Scale; MRI: Magnetic Resonance Imaging; BDI: Beck Depression Inventory; RRMS: Relapsing-Remitting Multiple Sclerosis; ARR: Annualized Relapse Rate; MSFC: Multiple Sclerosis Functional Composite; DMT: Disease Modifying Therapy, including interferon beta 1a and 1b (IFNβ-1a and 1b) and glatiramer acetate (GA); TSQM: Treatment Satisfaction Questionnaire for Medication; SF-36: 36-item Short-Form Health Survey; SDMT: Symbol Digit Modalities Test.

Fig. S1 (ARR)

Study or Subgroup	Experimental Mean	Experimental SD	Experimental Total	Control Mean	Control SD	Control Total	Weight	Mean Difference IV, Random, 95% CI	Year
**1.1.1 fingolimod 0.25mg**									
Cree 2020	0.22	0.49	366	0.26	0.55	324	100.0%	-0.04 [-0.12, 0.04]	2020
**Subtotal (95% CI)**			**366**			**324**	**100.0%**	**-0.04 [-0.12, 0.04]**	
Heterogeneity: Not applicable									
Test for overall effect: Z = 1.00 (P = 0.32)									
**1.1.2 fingolimod 0.5mg**									
Cohen 2010	0.16	0.42	429	0.33	0.74	431	18.9%	-0.17 [-0.25, -0.09]	2010
Kappos 2010	0.18	0.32	425	0.4	0.63	418	21.8%	-0.22 [-0.29, -0.15]	2010
Saida 2012	0.5	0.81	57	0.99	1.23	57	1.8%	-0.49 [-0.87, -0.11]	2012
Calabresi 2014	0.21	0.39	358	0.4	0.58	355	20.6%	-0.19 [-0.26, -0.12]	2014
Cree 2018	0.22	0.53	433	0.31	0.84	428	16.2%	-0.09 [-0.18, 0.00]	2018
Cree 2020	0.15	0.38	345	0.26	0.55	324	20.7%	-0.11 [-0.18, -0.04]	2020
**Subtotal (95% CI)**			**2047**			**2013**	**100.0%**	**-0.17 [-0.22, -0.11]**	
Heterogeneity: Tau² = 0.00; Chi² = 10.47, df = 5 (P = 0.06); I² = 52%									
Test for overall effect: Z = 6.22 (P < 0.00001)									
**1.1.3 fingolimod 1.25mg**									
Kappos 2010	0.16	0.32	429	0.4	0.63	418	47.6%	-0.24 [-0.31, -0.17]	2010
Saida 2012	0.41	0.71	54	0.99	1.23	57	4.6%	-0.58 [-0.95, -0.21]	2012
Calabresi 2014	0.2	0.29	370	0.4	0.58	355	47.8%	-0.20 [-0.27, -0.13]	2014
**Subtotal (95% CI)**			**853**			**830**	**100.0%**	**-0.24 [-0.32, -0.15]**	
Heterogeneity: Tau² = 0.00; Chi² = 4.23, df = 2 (P = 0.12); I² = 53%									
Test for overall effect: Z = 5.63 (P < 0.00001)									

Test for subgroup differences: Chi² = 12.18, df = 2 (P = 0.002), I² = 83.6%

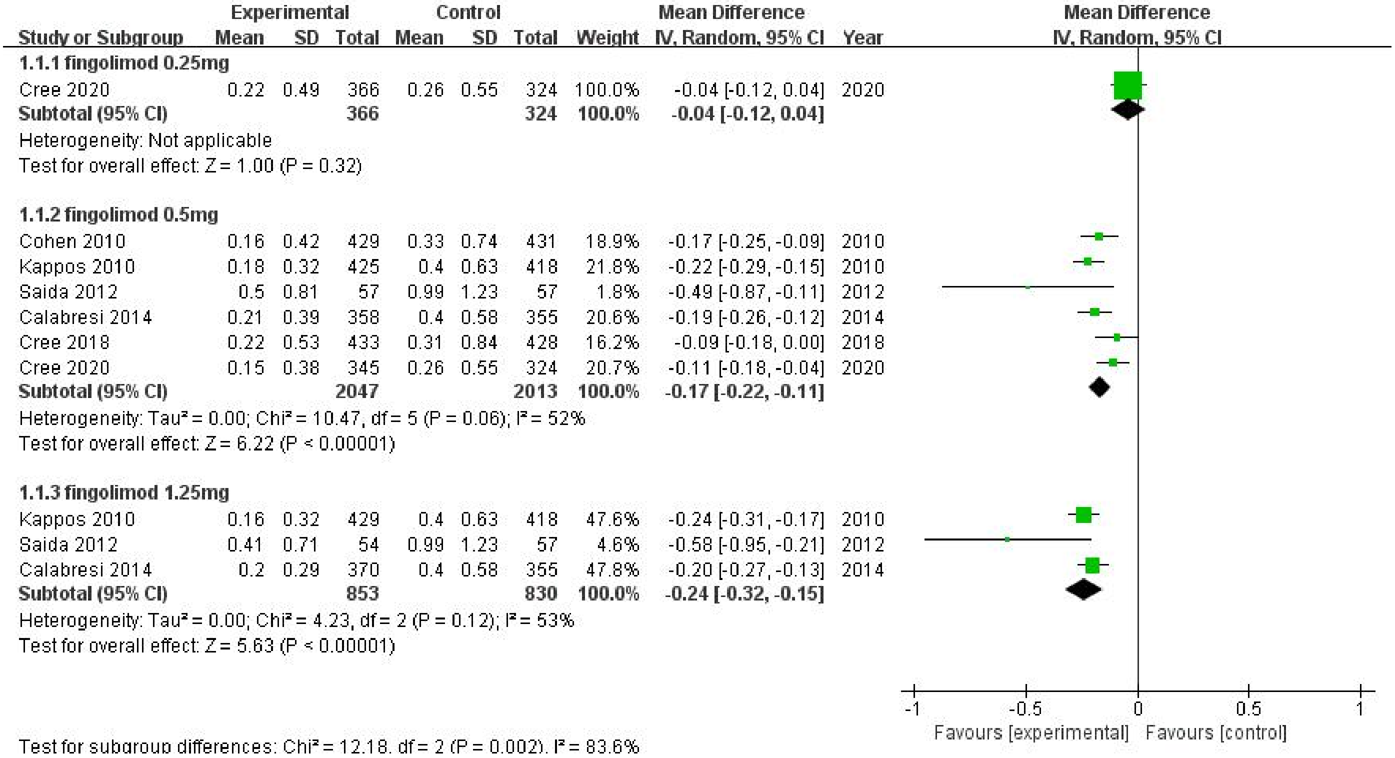

Fig. S2 (Number of patients free of relapse)

Study or Subgroup	Experimental Events	Experimental Total	Control Events	Control Total	Weight	Risk Ratio M-H, Random, 95% CI	Year
**2.1.1 fingolimod 0.25mg**							
Cree 2020	307	366	260	324	100.0%	1.05 [0.97, 1.12]	2020
**Subtotal (95% CI)**		**366**		**324**	**100.0%**	**1.05 [0.97, 1.12]**	
Total events	307		260				
Heterogeneity: Not applicable							
Test for overall effect: Z = 1.24 (P = 0.22)							
**2.1.2 fingolimod 0.5mg**							
Cohen 2010	354	429	302	431	27.2%	1.18 [1.09, 1.27]	2010
Saida 2012	45	57	37	57	10.8%	1.22 [0.96, 1.54]	2012
Calabresi 2014	256	358	187	355	21.6%	1.36 [1.21, 1.53]	2014
Comi 2017	88	104	32	47	12.3%	1.24 [1.01, 1.54]	2017
Cree 2020	299	345	260	324	28.1%	1.08 [1.01, 1.16]	2020
**Subtotal (95% CI)**		**1293**		**1214**	**100.0%**	**1.20 [1.09, 1.31]**	
Total events	1042		818				
Heterogeneity: Tau² = 0.01; Chi² = 12.98, df = 4 (P = 0.01); I² = 69%							
Test for overall effect: Z = 3.77 (P = 0.0002)							
**2.1.3 fingolimod 1.25mg**							
Kappos 2006	86	93	66	92	23.9%	1.29 [1.12, 1.48]	2006
Cohen 2010	338	420	302	431	34.2%	1.15 [1.06, 1.24]	2010
Saida 2012	45	54	37	57	14.3%	1.28 [1.03, 1.61]	2012
Calabresi 2014	271	370	187	355	27.7%	1.39 [1.24, 1.56]	2014
**Subtotal (95% CI)**		**937**		**935**	**100.0%**	**1.26 [1.14, 1.40]**	
Total events	740		592				
Heterogeneity: Tau² = 0.01; Chi² = 8.05, df = 3 (P = 0.05); I² = 63%							
Test for overall effect: Z = 4.40 (P < 0.0001)							
**2.1.4 fingolimod 5.0mg**							
Kappos 2006	86	92	66	92	100.0%	1.30 [1.13, 1.50]	2006
**Subtotal (95% CI)**		**92**		**92**	**100.0%**	**1.30 [1.13, 1.50]**	
Total events	86		66				
Heterogeneity: Not applicable							
Test for overall effect: Z = 3.73 (P = 0.0002)							

Test for subgroup differences: Chi² = 14.08, df = 3 (P = 0.003), I² = 78.7%

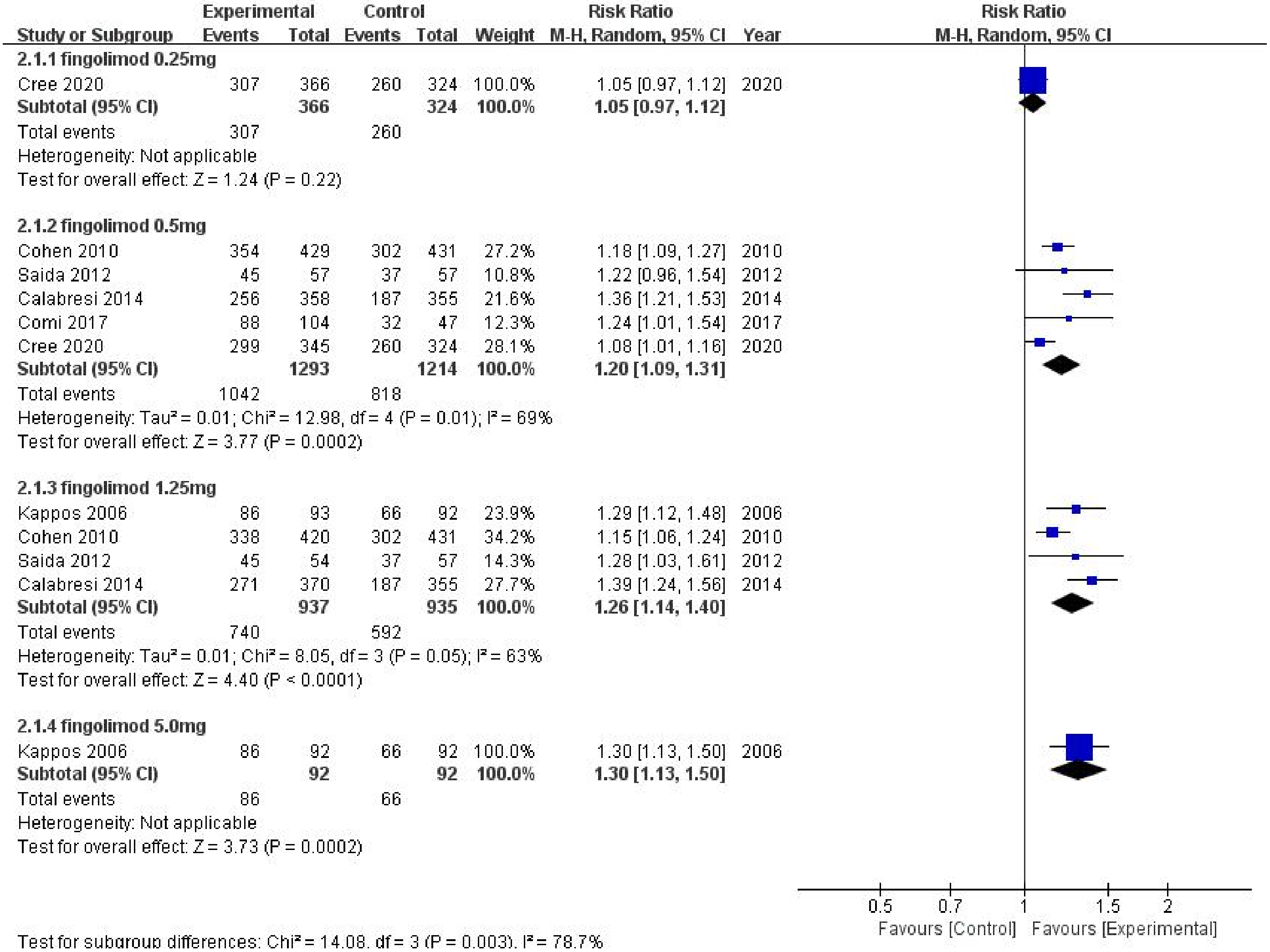

Fig. S3 (ΔEDSS)

Study or Subgroup	Experimental Mean	Experimental SD	Experimental Total	Control Mean	Control SD	Control Total	Weight	Mean Difference IV, Random, 95% CI	Year
**7.1.1 fingolimo 0.5mg**									
Kappos 2010	0	0.88	425	0.13	0.94	418	31.8%	-0.13 [-0.25, -0.01]	2010
Cohen 2010	-0.08	0.79	429	0.01	0.78	431	43.7%	-0.09 [-0.19, 0.01]	2010
Calabresi 2014	0.046	1.02	358	0.055	1.2	355	18.0%	-0.01 [-0.17, 0.15]	2014
Comi 2017	0.12	0.84	80	0.19	0.54	28	6.5%	-0.07 [-0.34, 0.20]	2017
**Subtotal (95% CI)**			**1292**			**1232**	**100.0%**	**-0.09 [-0.16, -0.02]**	
Heterogeneity: Tau² = 0.00; Chi² = 1.36, df = 3 (P = 0.71); I² = 0%									
Test for overall effect: Z = 2.45 (P = 0.01)									
**7.1.2 fingolimod 1.25mg**									
Kappos 2010	-0.03	0.88	429	0.13	0.94	418	37.1%	-0.16 [-0.28, -0.04]	2010
Cohen 2010	-0.11	0.9	420	0.01	0.78	431	43.5%	-0.12 [-0.23, -0.01]	2010
Calabresi 2014	-0.084	1.13	370	0.055	1.2	355	19.4%	-0.14 [-0.31, 0.03]	2014
**Subtotal (95% CI)**			**1219**			**1204**	**100.0%**	**-0.14 [-0.21, -0.06]**	
Heterogeneity: Tau² = 0.00; Chi² = 0.22, df = 2 (P = 0.90); I² = 0%									
Test for overall effect: Z = 3.63 (P = 0.0003)									

Test for subgroup differences: Chi² = 0.99, df = 1 (P = 0.32), I² = 0%

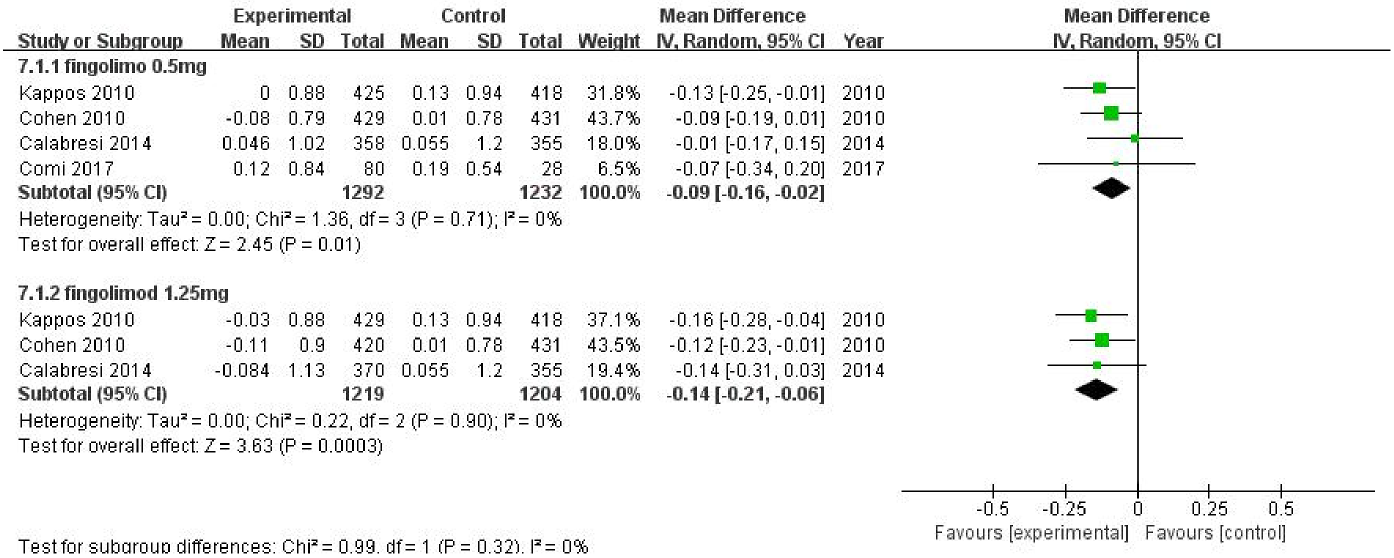

Fig. S4 (Number of patients free of increased gadolinium-enhanced lesions in T1)

Study or Subgroup	Experimental Events	Experimental Total	Control Events	Control Total	Weight	Risk Ratio M-H, Random, 95% CI	Year
**3.1.1 fingolimod 0.25mg**							
Cree 2020	269	331	202	272	100.0%	1.09 [1.00, 1.19]	2020
**Subtotal (95% CI)**		**331**		**272**	**100.0%**	**1.09 [1.00, 1.19]**	
Total events	269		202				
Heterogeneity: Not applicable							
Test for overall effect: Z = 2.03 (P = 0.04)							
**3.1.2 fingolimod 0.5mg**							
Cohen 2010	337	374	286	354	24.1%	1.12 [1.05, 1.19]	2010
Kappos 2010	331	369	216	332	22.3%	1.38 [1.27, 1.50]	2010
Saida 2012	39	45	29	50	9.9%	1.49 [1.15, 1.94]	2012
Calabresi 2014	234	269	167	256	21.1%	1.33 [1.21, 1.47]	2014
Cree 2020	261	303	202	272	22.5%	1.16 [1.07, 1.26]	2020
**Subtotal (95% CI)**		**1360**		**1264**	**100.0%**	**1.26 [1.14, 1.40]**	
Total events	1202		900				
Heterogeneity: Tau² = 0.01; Chi² = 23.97, df = 4 (P < 0.0001); I² = 83%							
Test for overall effect: Z = 4.34 (P < 0.0001)							
**3.1.3 fingolimod 1.25mg**							
Kappos 2006	64	83	38	81	14.8%	1.64 [1.27, 2.13]	2006
Kappos 2010	308	343	216	332	23.0%	1.38 [1.27, 1.50]	2010
Cohen 2010	321	352	286	354	23.8%	1.13 [1.06, 1.20]	2010
Saida 2012	47	48	29	50	15.7%	1.69 [1.33, 2.14]	2012
Calabresi 2014	242	251	167	256	22.7%	1.48 [1.35, 1.62]	2014
**Subtotal (95% CI)**		**1077**		**1073**	**100.0%**	**1.42 [1.21, 1.66]**	
Total events	982		736				
Heterogeneity: Tau² = 0.03; Chi² = 40.57, df = 4 (P < 0.00001); I² = 90%							
Test for overall effect: Z = 4.33 (P < 0.0001)							
**3.1.4 fingolimod 5.0mg**							
Kappos 2006	63	77	38	81	100.0%	1.74 [1.35, 2.25]	2006
**Subtotal (95% CI)**		**77**		**81**	**100.0%**	**1.74 [1.35, 2.25]**	
Total events	63		38				
Heterogeneity: Not applicable							
Test for overall effect: Z = 4.28 (P < 0.0001)							

Test for subgroup differences: Chi² = 17.38, df = 3 (P = 0.0006), I² = 82.7%

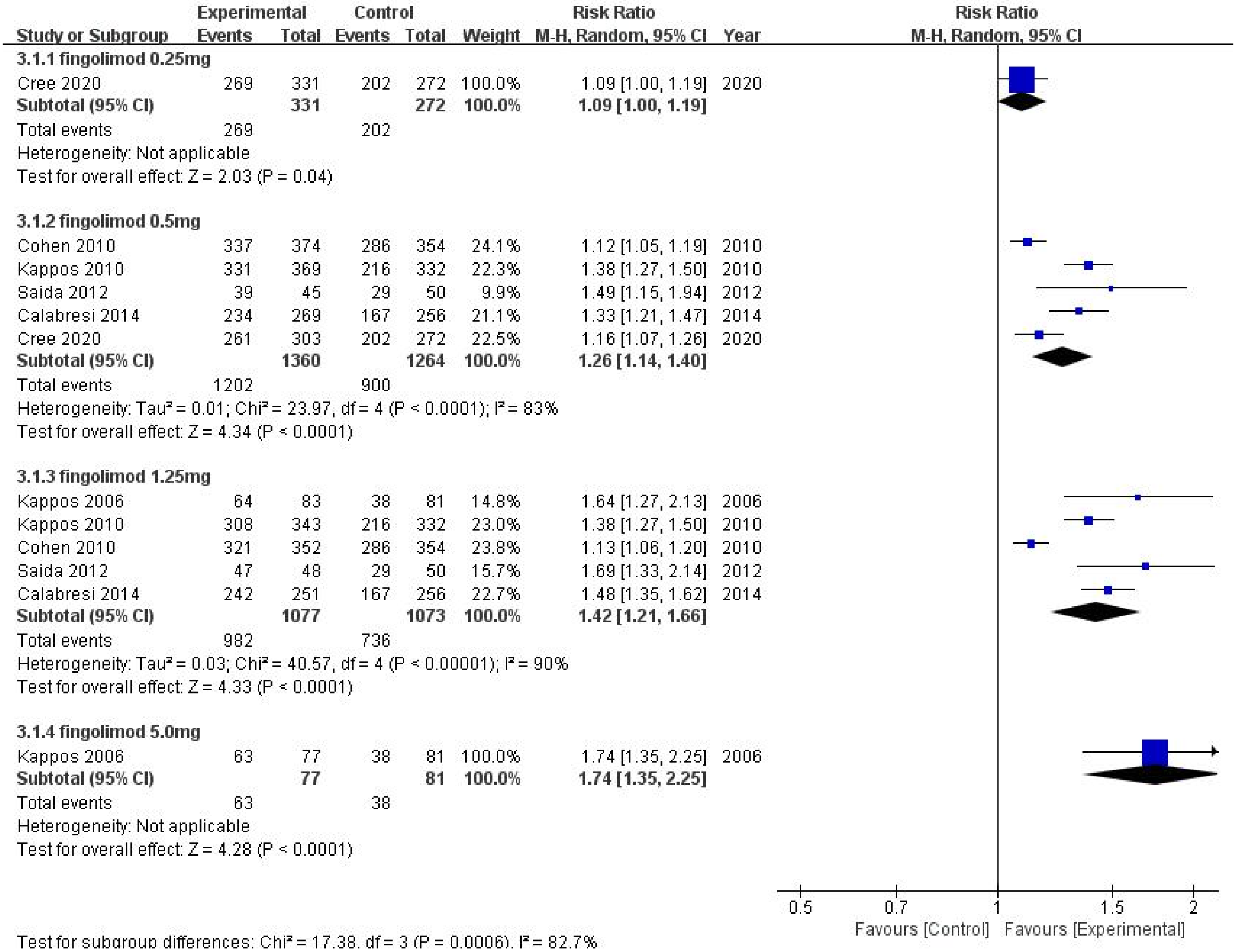

Fig. S5 (Number of patients with no new or newly enlarged lesions in T2)

Study or Subgroup	Experimental Events	Experimental Total	Control Events	Control Total	Weight	Risk Ratio M-H, Random, 95% CI	Year
**4.1.1 fingolimod 0.25mg**							
Cree 2020	155	331	96	272	100.0%	1.33 [1.09, 1.62]	2020
**Subtotal (95% CI)**		**331**		**272**	**100.0%**	**1.33 [1.09, 1.62]**	
Total events	155		96				
Heterogeneity: Not applicable							
Test for overall effect: Z = 2.80 (P = 0.005)							
**4.1.2 fingolimod 0.5mg**							
Cohen 2010	204	372	165	361	22.3%	1.20 [1.04, 1.39]	2010
Kappos 2010	187	370	72	339	20.6%	2.38 [1.89, 2.99]	2010
Saida 2012	31	48	18	50	15.6%	1.79 [1.17, 2.74]	2012
Calabresi 2014	133	264	65	251	20.3%	1.95 [1.53, 2.48]	2014
Cree 2020	156	345	96	272	21.3%	1.28 [1.05, 1.56]	2020
**Subtotal (95% CI)**		**1399**		**1273**	**100.0%**	**1.64 [1.23, 2.19]**	
Total events	711		416				
Heterogeneity: Tau² = 0.09; Chi² = 33.13, df = 4 (P < 0.00001); I² = 88%							
Test for overall effect: Z = 3.39 (P = 0.0007)							
**4.1.3 fingolimod 1.25mg**							
Cohen 2010	168	350	165	361	26.4%	1.05 [0.90, 1.23]	2010
Kappos 2010	175	337	72	339	25.6%	2.44 [1.94, 3.08]	2010
Saida 2012	28	48	18	50	22.4%	1.62 [1.04, 2.52]	2012
Calabresi 2014	155	245	65	251	25.6%	2.44 [1.94, 3.07]	2014
**Subtotal (95% CI)**		**980**		**1001**	**100.0%**	**1.78 [1.08, 2.96]**	
Total events	526		320				
Heterogeneity: Tau² = 0.25; Chi² = 55.10, df = 3 (P < 0.00001); I² = 95%							
Test for overall effect: Z = 2.24 (P = 0.02)							

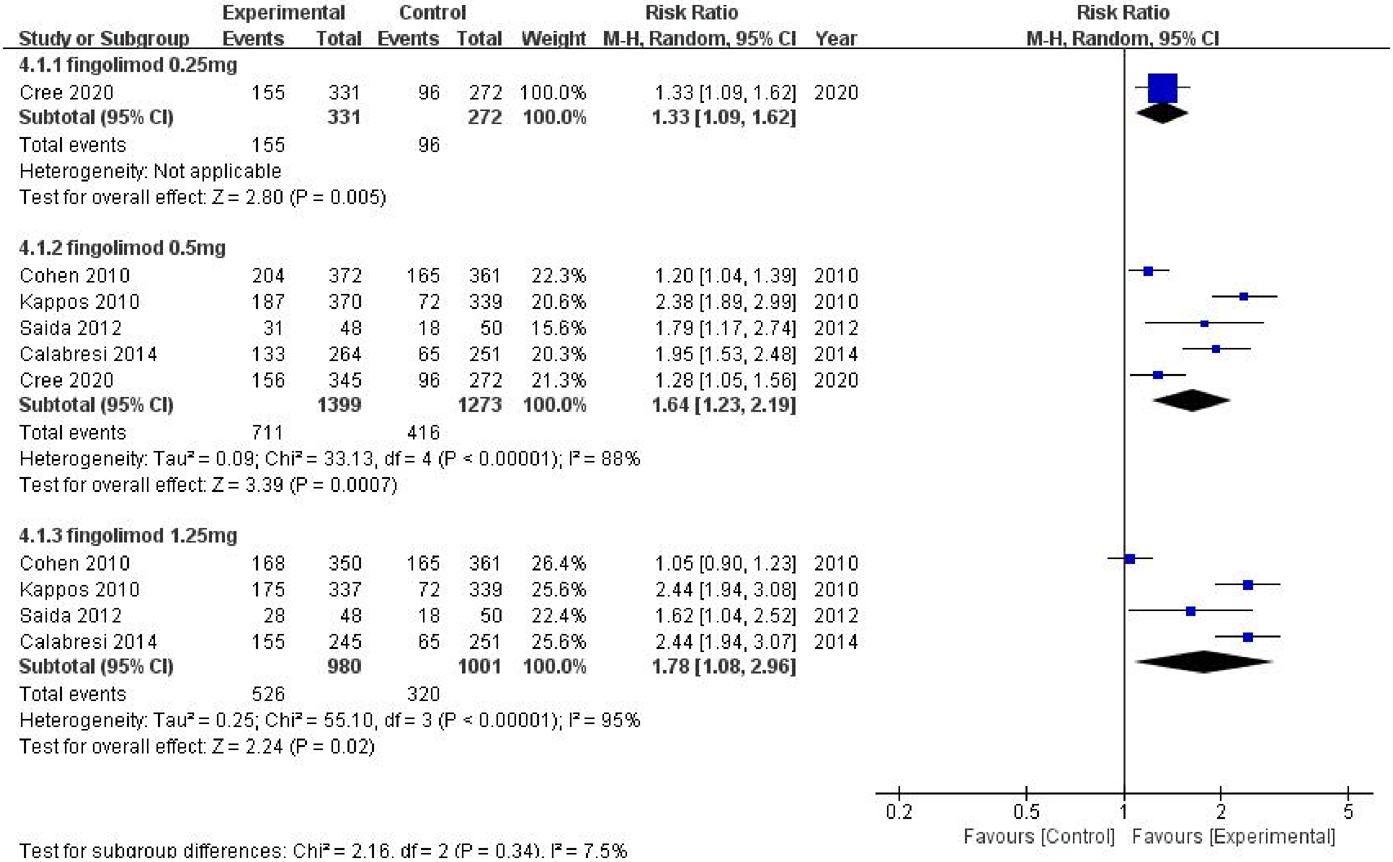

Test for subgroup differences: Chi² = 2.16, df = 2 (P = 0.34), I² = 7.5%

Fig. S6 (PBVC)

Study or Subgroup	Experimental Mean	Experimental SD	Experimental Total	Control Mean	Control SD	Control Total	Weight	Mean Difference IV, Random, 95% CI	Year
**5.1.1 fingolimod 0.25mg**									
Cree 2020	-0.64	0.81	305	-0.56	0.78	252	100.0%	-0.08 [-0.21, 0.05]	2020
**Subtotal (95% CI)**			**305**			**252**	**100.0%**	**-0.08 [-0.21, 0.05]**	
Heterogeneity: Not applicable									
Test for overall effect: Z = 1.18 (P = 0.24)									
**5.1.2 fingolimod 0.5mg**									
Cohen 2010	-0.31	0.65	429	-0.45	0.73	431	20.2%	0.14 [0.05, 0.23]	2010
Kappos 2010	-0.84	1.31	357	-1.31	1.5	331	16.3%	0.47 [0.26, 0.68]	2010
Calabresi 2014	-0.858	1.222	266	-1.279	1.503	249	15.3%	0.42 [0.18, 0.66]	2014
Comi 2017	-0.6	0.83	80	-0.96	0.71	28	12.4%	0.36 [0.04, 0.68]	2017
Cree 2018	-0.48	1.11	370	-0.75	1.32	246	16.7%	0.27 [0.07, 0.47]	2018
Cree 2020	-0.65	0.78	284	-0.56	0.78	252	19.1%	-0.09 [-0.22, 0.04]	2020
**Subtotal (95% CI)**			**1786**			**1537**	**100.0%**	**0.24 [0.07, 0.42]**	
Heterogeneity: Tau² = 0.04; Chi² = 29.48, df = 5 (P < 0.0001); I² = 83%									
Test for overall effect: Z = 2.73 (P = 0.006)									
**5.1.3 fingolimod 1.25mg**									
Kappos 2006	-0.22	2.04	83	-0.31	3.55	81	8.4%	0.09 [-0.80, 0.98]	2006
Kappos 2010	-0.89	1.39	334	-1.31	1.5	331	29.5%	0.42 [0.20, 0.64]	2010
Cohen 2010	-0.3	0.65	420	-0.45	0.73	431	34.2%	0.15 [0.06, 0.24]	2010
Calabresi 2014	-0.595	1.39	247	-1.279	1.503	249	27.9%	0.68 [0.43, 0.94]	2014
**Subtotal (95% CI)**			**1084**			**1092**	**100.0%**	**0.37 [0.08, 0.67]**	
Heterogeneity: Tau² = 0.06; Chi² = 17.97, df = 3 (P = 0.0004); I² = 83%									
Test for overall effect: Z = 2.49 (P = 0.01)									
**5.1.4 fingolimod 5.0mg**									
Kappos 2006	-0.4	2.19	77	-0.31	3.55	81	100.0%	-0.09 [-1.00, 0.82]	2006
**Subtotal (95% CI)**			**77**			**81**	**100.0%**	**-0.09 [-1.00, 0.82]**	
Heterogeneity: Not applicable									
Test for overall effect: Z = 0.19 (P = 0.85)									

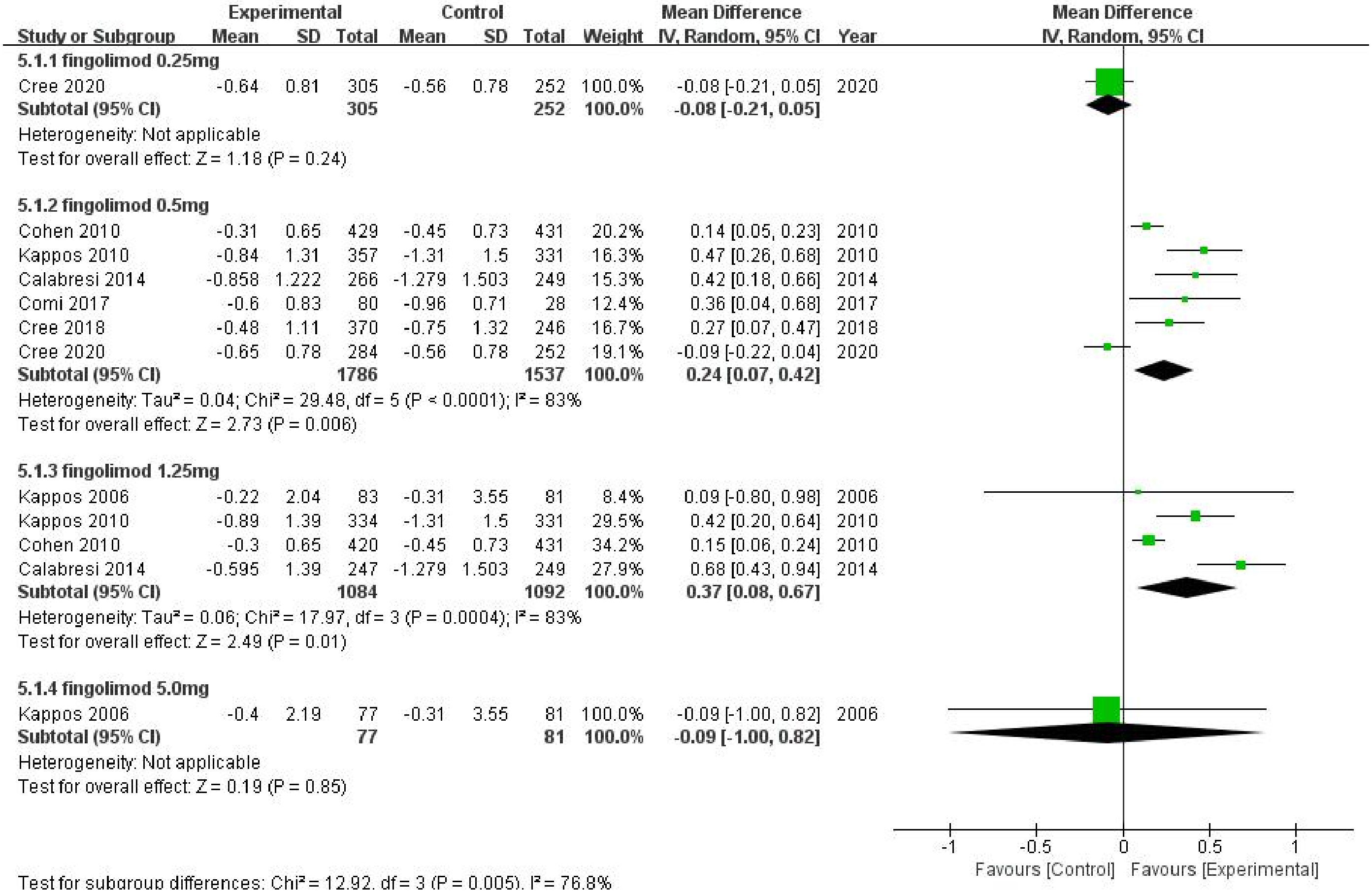

Test for subgroup differences: Chi² = 12.92, df = 3 (P = 0.005), I² = 76.8%

Fig. S7 (ΔBDI)

Study or Subgroup	Experimental Mean	Experimental SD	Experimental Total	Control Mean	Control SD	Control Total	Weight	Mean Difference IV, Random, 95% CI
**6.1.1 fingolimod 0.5mg**								
Comi 2017	-0.68	7.57	80	0.3	5.63	28	8.8%	-0.98 [-3.64, 1.68]
Fox 2014	-3.2	0.49	768	-0.8	0.49	240	65.4%	-2.40 [-2.47, -2.33]
NCT01317004	-1.15	3.59	48	-0.12	3.06	11	13.4%	-1.03 [-3.10, 1.04]
NCT01534182	-2.88	6.8	229	-1.86	8.04	62	12.3%	-1.02 [-3.21, 1.17]
**Subtotal (95% CI)**			**1125**			**341**	**100.0%**	**-1.92 [-2.77, -1.07]**

Heterogeneity: Tau² = 0.29; Chi² = 4.28, df = 3 (P = 0.23); I² = 30%
Test for overall effect: Z = 4.42 (P < 0.00001)

Test for subgroup differences: Not applicable

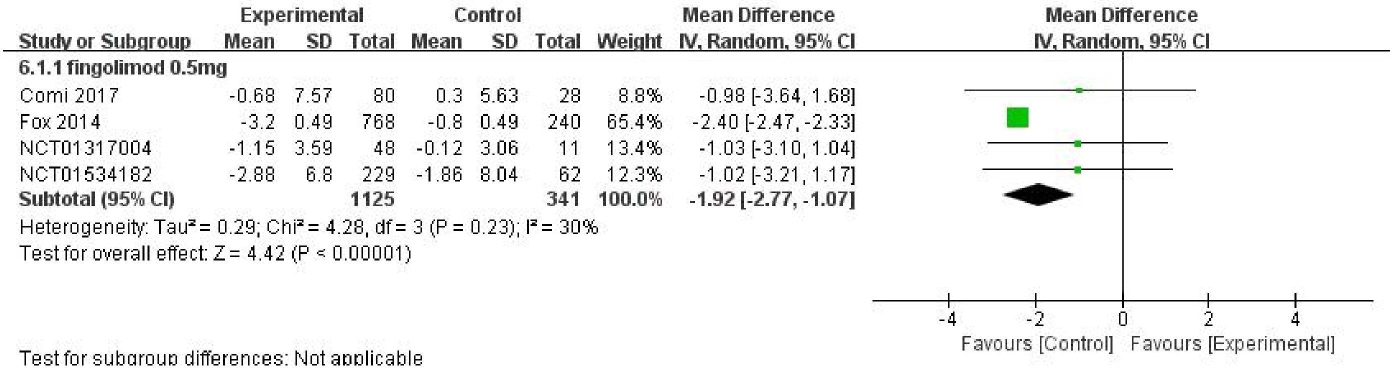

Fig. S8 (ΔTSQM)

Study or Subgroup	Experimental Mean	Experimental SD	Experimental Total	Control Mean	Control SD	Control Total	Weight	Mean Difference IV, Random, 95% CI
**8.1.1 fingolimod 0.25mg**								
Cree 2020	20.5	32.2	201	9.4	30.4	180	100.0%	11.10 [4.81, 17.39]
**Subtotal (95% CI)**			**201**			**180**	**100.0%**	**11.10 [4.81, 17.39]**
Heterogeneity: Not applicable								
Test for overall effect: Z = 3.46 (P = 0.0005)								
**8.1.2 fingolimod 0.5mg**								
Cree 2020	19.2	31.9	185	9.4	30.4	180	28.4%	9.80 [3.41, 16.19]
Fox 2014	20.4	42.5272	767	2.9	25.9722	239	38.1%	17.50 [13.04, 21.96]
NCT01317004	19.57	21	46	5.83	16.47	10	12.8%	13.74 [1.86, 25.62]
NCT01534182	22.69	28.12	229	13.92	30.63	62	20.7%	8.77 [0.32, 17.22]
**Subtotal (95% CI)**			**1227**			**491**	**100.0%**	**13.03 [8.20, 17.85]**

Heterogeneity: Tau² = 10.72; Chi² = 5.50, df = 3 (P = 0.14); I² = 45%
Test for overall effect: Z = 5.29 (P < 0.00001)

Test for subgroup differences: Chi² = 0.23, df = 1 (P = 0.63), I² = 0%

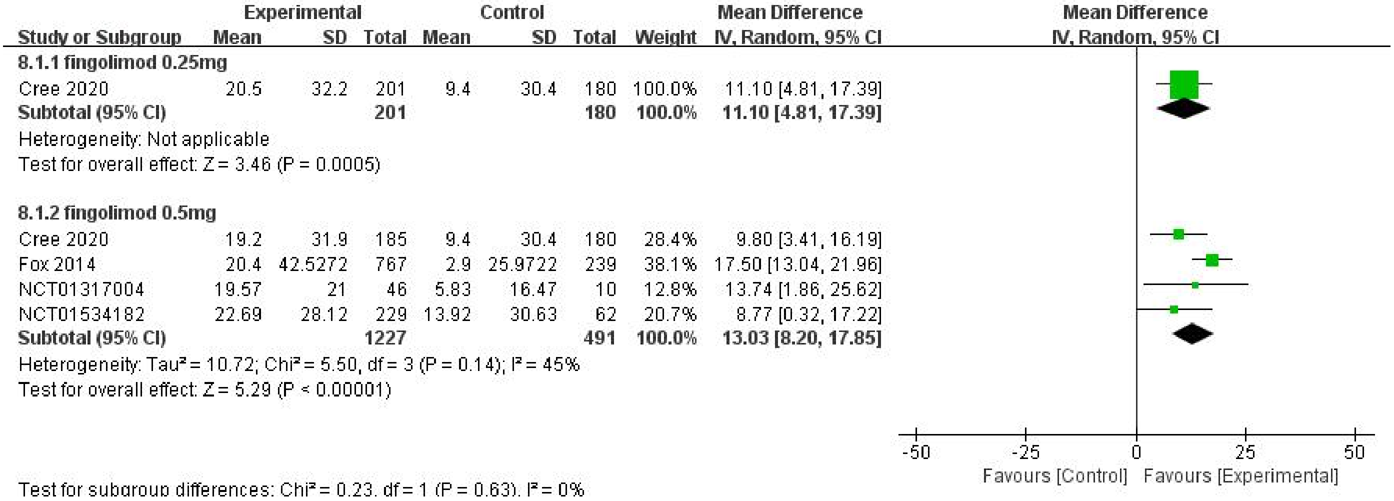

Fig. S9 (AEs)

Study or Subgroup	Experimental Events	Experimental Total	Control Events	Control Total	Weight	Risk Ratio M-H, Random, 95% CI	Year
**11.1.1 fingolimod 0.25mg**							
Cree 2020	323	366	283	324	100.0%	1.01 [0.96, 1.07]	2020
**Subtotal (95% CI)**		**366**		**324**	**100.0%**	**1.01 [0.96, 1.07]**	
Total events	323		283				
Heterogeneity: Not applicable							
Test for overall effect: Z = 0.36 (P = 0.72)							
**11.1.2 fingolimod 0.5mg**							
Kappos 2010	401	425	387	418	15.5%	1.02 [0.98, 1.06]	2010
Cohen 2010	369	429	395	431	14.8%	0.94 [0.89, 0.98]	2010
Saida 2012	52	57	45	57	7.3%	1.16 [0.99, 1.35]	2012
Fox 2014	617	783	152	245	10.6%	1.27 [1.14, 1.41]	2014
NCT01534182	73	230	26	64	2.2%	0.78 [0.55, 1.11]	2014
Calabresi 2014	350	358	343	355	16.0%	1.01 [0.99, 1.04]	2014
NCT01317004	13	50	7	11	0.7%	0.41 [0.21, 0.78]	2015
Comi 2017	83	104	28	47	3.8%	1.34 [1.04, 1.73]	2017
Cree 2018	394	433	355	428	14.5%	1.10 [1.04, 1.16]	2018
Cree 2020	312	345	283	324	14.4%	1.04 [0.98, 1.09]	2020
**Subtotal (95% CI)**		**3214**		**2380**	**100.0%**	**1.05 [0.99, 1.11]**	
Total events	2664		2021				
Heterogeneity: Tau² = 0.01; Chi² = 61.46, df = 9 (P < 0.00001); I² = 85%							
Test for overall effect: Z = 1.69 (P = 0.09)							
**11.1.3 fingolimod 1.25mg**							
Kappos 2006	79	94	76	93	4.3%	1.03 [0.90, 1.17]	2006
Kappos 2010	404	429	387	418	29.5%	1.02 [0.98, 1.05]	2010
Cohen 2010	380	420	395	431	25.0%	0.99 [0.95, 1.03]	2010
Saida 2012	51	54	45	57	3.4%	1.20 [1.03, 1.39]	2012
Calabresi 2014	359	370	343	355	37.8%	1.00 [0.98, 1.03]	2014
**Subtotal (95% CI)**		**1367**		**1354**	**100.0%**	**1.01 [0.98, 1.04]**	
Total events	1273		1246				
Heterogeneity: Tau² = 0.00; Chi² = 6.57, df = 4 (P = 0.16); I² = 39%							
Test for overall effect: Z = 0.74 (P = 0.46)							
**11.1.4 fingolimod 5.0mg**							
Kappos 2006	90	94	76	93	100.0%	1.17 [1.05, 1.30]	2006
**Subtotal (95% CI)**		**94**		**93**	**100.0%**	**1.17 [1.05, 1.30]**	
Total events	90		76				
Heterogeneity: Not applicable							
Test for overall effect: Z = 2.95 (P = 0.003)							

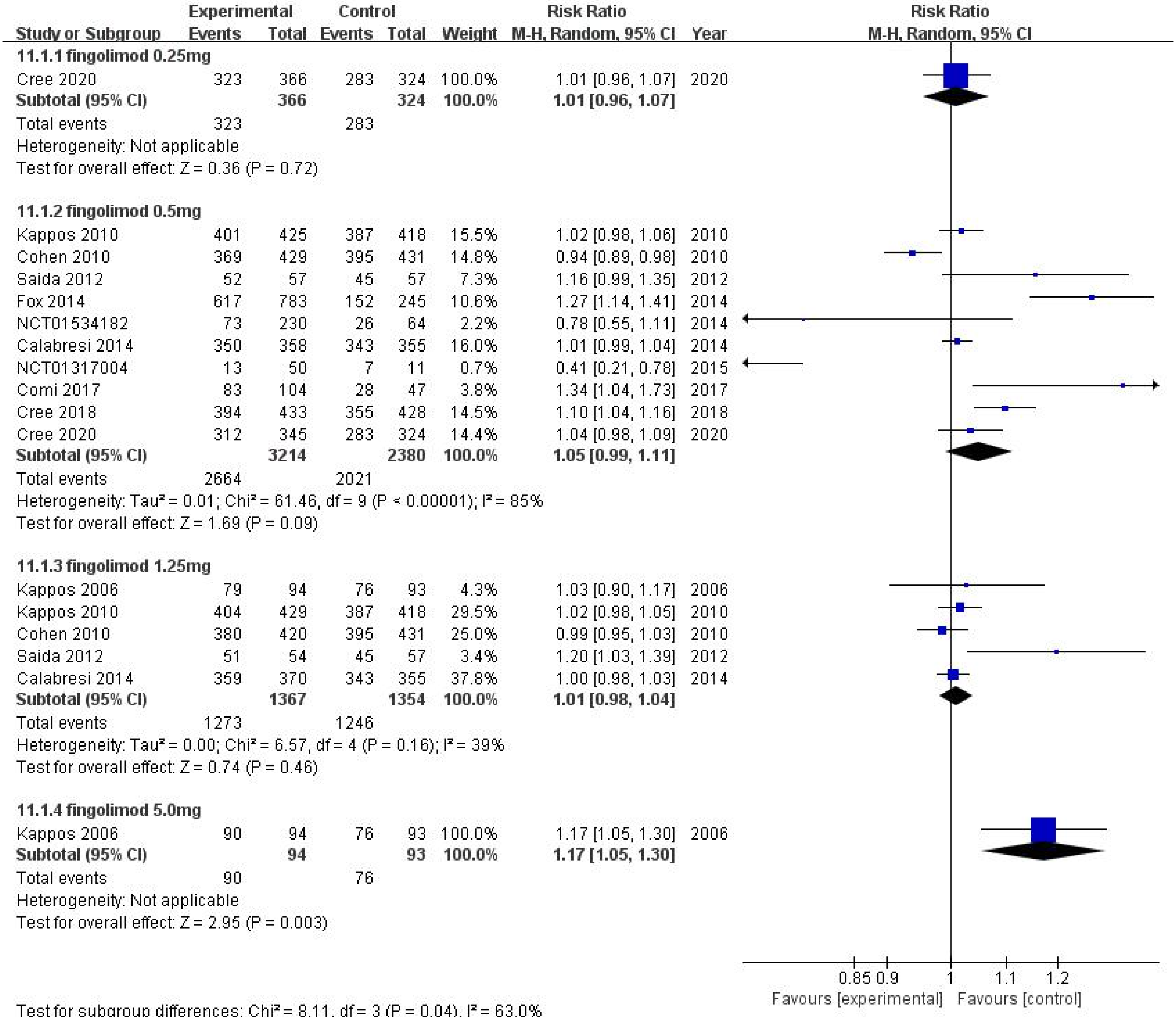

Test for subgroup differences: Chi² = 8.11, df = 3 (P = 0.04), I² = 63.0%

Fig. S10 (SAEs)

Study or Subgroup	Experimental Events	Experimental Total	Control Events	Control Total	Weight	Risk Ratio M-H, Random, 95% CI	Year
**12.1.1 fingolimod 0.25mg**							
Cree 2020	32	366	20	324	100.0%	1.42 [0.83, 2.43]	2020
**Subtotal (95% CI)**		**366**		**324**	**100.0%**	**1.42 [0.83, 2.43]**	
Total events	32		20				
Heterogeneity: Not applicable							
Test for overall effect: Z = 1.27 (P = 0.20)							
**12.1.2 fingolimod 0.5mg**							
Cohen 2010	30	429	25	431	17.1%	1.21 [0.72, 2.02]	2010
Kappos 2010	20	425	24	418	13.5%	0.82 [0.46, 1.46]	2010
Saida 2012	5	57	3	57	2.4%	1.67 [0.42, 6.65]	2012
Calabresi 2014	53	358	45	355	33.1%	1.17 [0.81, 1.69]	2014
Fox 2014	31	783	5	245	5.2%	1.94 [0.76, 4.93]	2014
NCT01534182	2	230	0	64	0.5%	1.41 [0.07, 28.94]	2014
NCT01317004	4	50	1	11	1.0%	0.88 [0.11, 7.13]	2015
Comi 2017	9	104	1	47	1.1%	4.07 [0.53, 31.19]	2017
Cree 2018	29	433	15	428	12.2%	1.91 [1.04, 3.51]	2018
Cree 2020	25	345	20	324	14.0%	1.17 [0.67, 2.07]	2020
**Subtotal (95% CI)**		**3214**		**2380**	**100.0%**	**1.25 [1.01, 1.54]**	
Total events	208		139				
Heterogeneity: Tau² = 0.00; Chi² = 6.57, df = 9 (P = 0.68); I² = 0%							
Test for overall effect: Z = 2.03 (P = 0.04)							
**12.1.3 fingolimod 1.25mg**							
Kappos 2006	8	94	6	93	10.1%	1.32 [0.48, 3.65]	2006
Cohen 2010	45	420	25	431	27.0%	1.85 [1.15, 2.96]	2010
Kappos 2010	23	429	24	418	22.9%	0.93 [0.54, 1.63]	2010
Saida 2012	11	54	3	57	7.5%	3.87 [1.14, 13.12]	2012
Calabresi 2014	53	370	45	355	32.5%	1.13 [0.78, 1.64]	2014
**Subtotal (95% CI)**		**1367**		**1354**	**100.0%**	**1.38 [0.96, 1.98]**	
Total events	140		103				
Heterogeneity: Tau² = 0.07; Chi² = 7.13, df = 4 (P = 0.13); I² = 44%							
Test for overall effect: Z = 1.72 (P = 0.09)							
**12.1.4 fingolimod 5.0mg**							
Kappos 2006	13	94	6	93	100.0%	2.14 [0.85, 5.40]	2006
**Subtotal (95% CI)**		**94**		**93**	**100.0%**	**2.14 [0.85, 5.40]**	
Total events	13		6				
Heterogeneity: Not applicable							
Test for overall effect: Z = 1.62 (P = 0.11)							

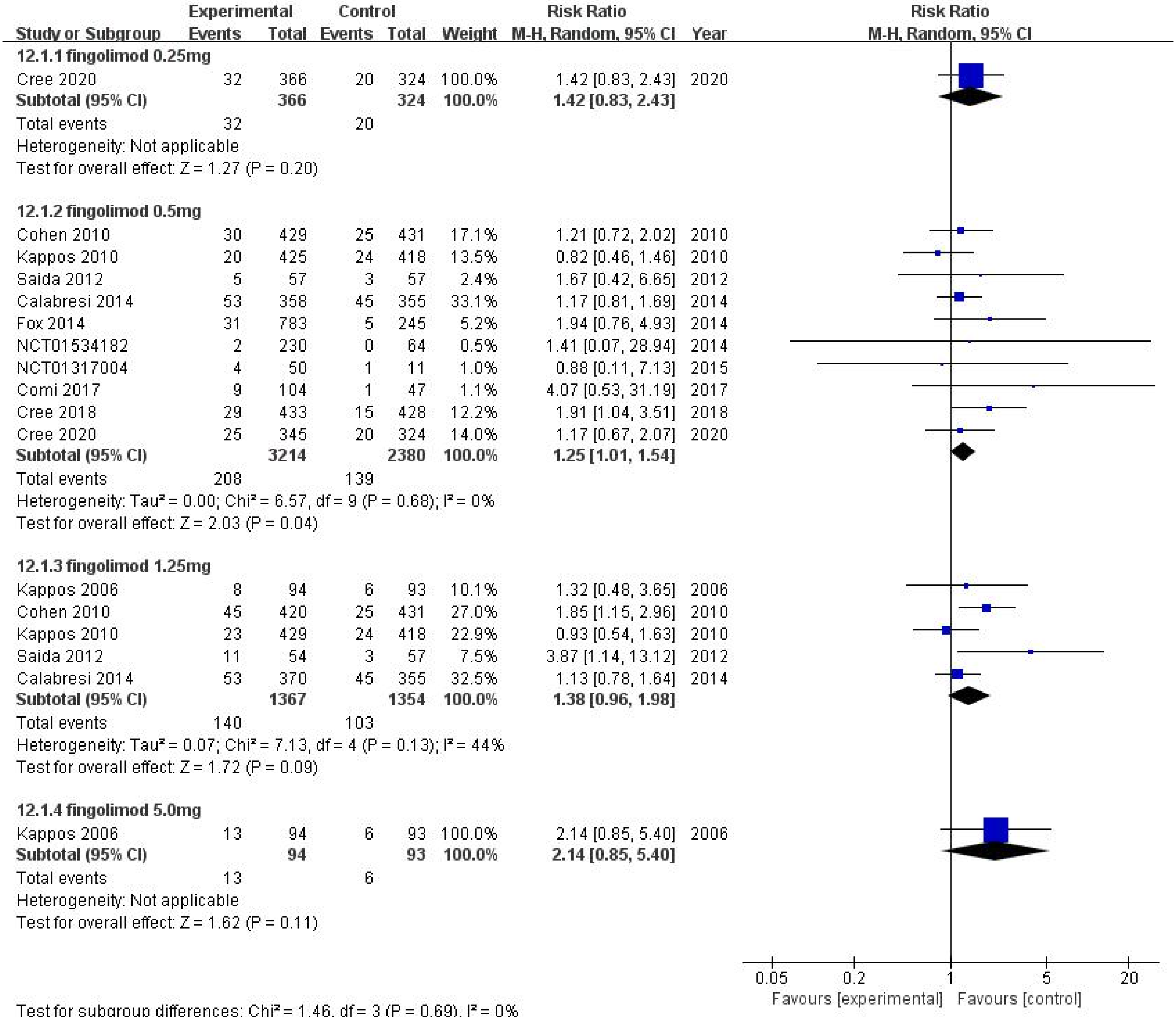

Test for subgroup differences: Chi² = 1.46, df = 3 (P = 0.69), I² = 0%

Fig. S11 (Sub-analysis of specific SAEs in the fingolimod 0.5 mg/d group)

A. Bradycardia

Study or Subgroup	Experimental Events	Experimental Total	Control Events	Control Total	Weight	Risk Ratio M-H, Random, 95% CI	Year
Kappos 2010	2	429	0	431	20.5%	5.02 [0.24, 104.32]	2010
Cohen 2010	4	425	1	418	39.4%	3.93 [0.44, 35.05]	2010
Saida 2012	3	57	0	57	21.8%	7.00 [0.37, 132.51]	2012
Calabresi 2014	0	358	1	355	18.4%	0.33 [0.01, 8.09]	2014
**Total (95% CI)**		**1269**		**1261**	**100.0%**	**2.97 [0.75, 11.72]**	
Total events	9		2				

Heterogeneity: Tau² = 0.00; Chi² = 2.32, df = 3 (P = 0.51); I² = 0%
Test for overall effect: Z = 1.56 (P = 0.12)

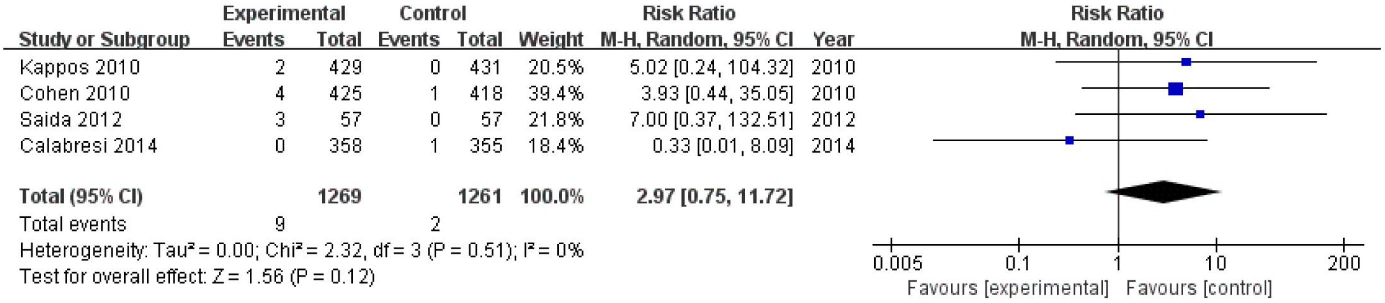

B. Atrioventricular block

Study or Subgroup	Experimental Events	Experimental Total	Control Events	Control Total	Weight	Risk Ratio M-H, Random, 95% CI	Year
Cohen 2010	2	429	0	431	25.0%	5.02 [0.24, 104.32]	2010
Saida 2012	3	57	2	57	75.0%	1.50 [0.26, 8.64]	2012
Calabresi 2014	0	358	0	355		Not estimable	2014
**Total (95% CI)**		**844**		**843**	**100.0%**	**2.03 [0.45, 9.25]**	
Total events	5		2				

Heterogeneity: Tau² = 0.00; Chi² = 0.47, df = 1 (P = 0.49); I² = 0%
Test for overall effect: Z = 0.91 (P = 0.36)

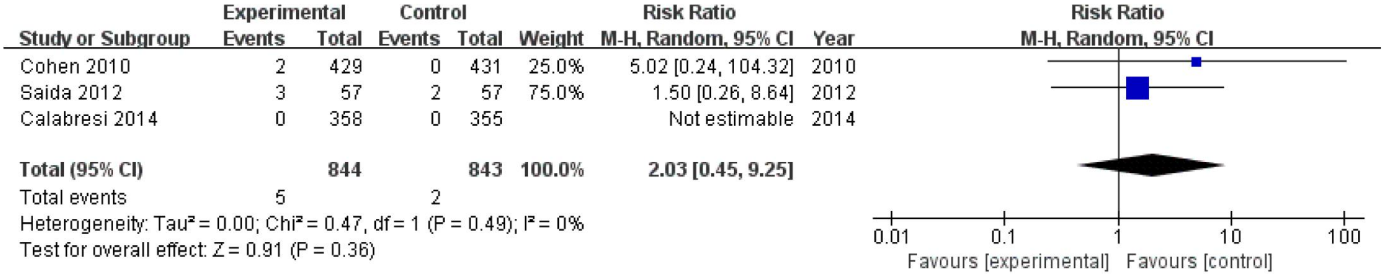

C. Chest pain

Study or Subgroup	Experimental Events	Experimental Total	Control Events	Control Total	Weight	Risk Ratio M-H, Random, 95% CI	Year
Kappos 2010	4	425	2	418	61.6%	1.97 [0.36, 10.68]	2010
Calabresi 2014	2	358	0	355	19.2%	4.96 [0.24, 102.91]	2014
Fox 2014	2	783	0	245	19.2%	1.57 [0.08, 32.57]	2014
**Total (95% CI)**		**1566**		**1018**	**100.0%**	**2.25 [0.60, 8.49]**	
Total events	8		2				

Heterogeneity: Tau² = 0.00; Chi² = 0.34, df = 2 (P = 0.84); I² = 0%
Test for overall effect: Z = 1.20 (P = 0.23)

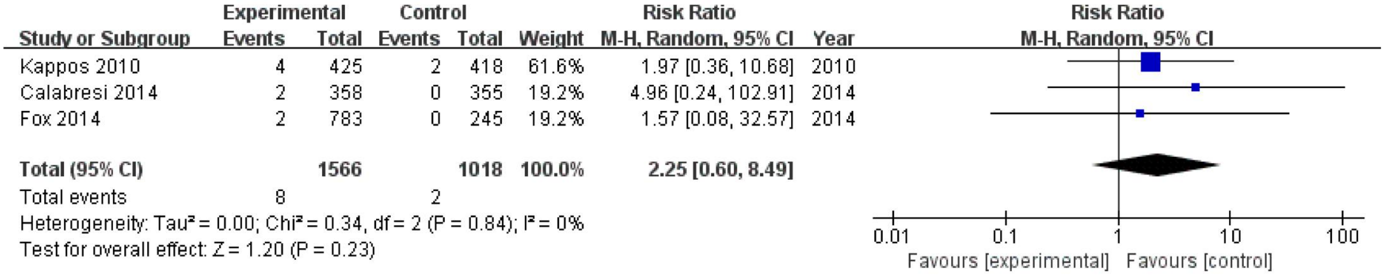

D. Dyspnea

Study or Subgroup	Experimental Events	Experimental Total	Control Events	Control Total	Weight	Risk Ratio M-H, Random, 95% CI
Cohen 2010	0	429	0	431		Not estimable
Cree 2020	2	345	0	324	100.0%	4.70 [0.23, 97.46]
**Total (95% CI)**		**774**		**755**	**100.0%**	**4.70 [0.23, 97.46]**
Total events	2		0			

Heterogeneity: Not applicable
Test for overall effect: Z = 1.00 (P = 0.32)

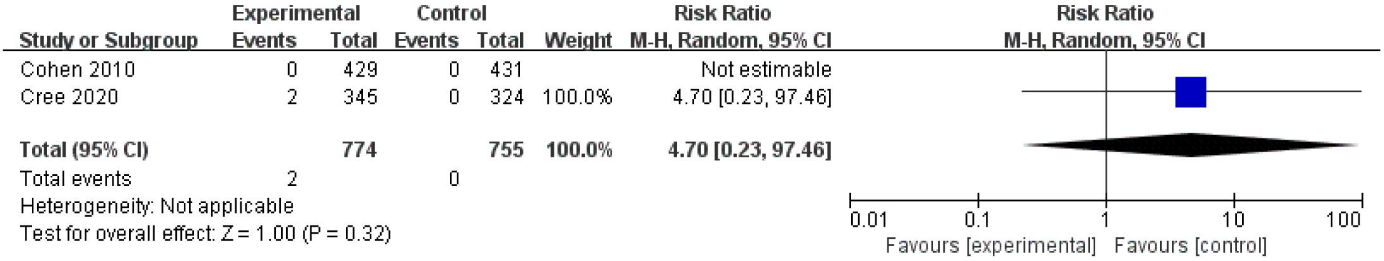

E. Infection

Study or Subgroup	Experimental Events	Experimental Total	Control Events	Control Total	Weight	Risk Ratio M-H, Random, 95% CI	Year
Kappos 2010	2	425	0	418	10.8%	4.92 [0.24, 102.13]	2010
Cohen 2010	1	429	3	431	16.4%	0.33 [0.03, 3.21]	2010
Calabresi 2014	11	358	4	355	31.8%	2.73 [0.88, 8.48]	2014
NCT01317004	0	50	1	11	10.2%	0.08 [0.00, 1.81]	2015
Cree 2018	3	433	0	428	11.2%	6.92 [0.36, 133.55]	2018
Cree 2020	2	345	2	324	19.6%	0.94 [0.13, 6.63]	2020
**Total (95% CI)**		**2040**		**1967**	**100.0%**	**1.29 [0.41, 4.02]**	
Total events	19		10				

Heterogeneity: Tau² = 0.72; Chi² = 7.99, df = 5 (P = 0.16); I² = 37%
Test for overall effect: Z = 0.44 (P = 0.66)

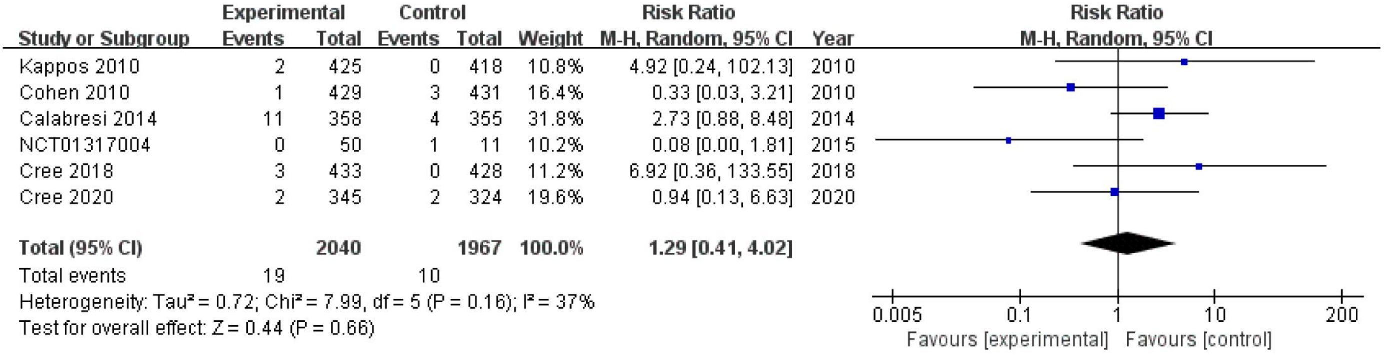

Fig. S12 (Sub-analysis of specific SAEs in the fingolimod 0.5 mg/d group)

A. MS relapse

Study or Subgroup	Experimental Events	Experimental Total	Control Events	Control Total	Weight	Risk Ratio M-H, Random, 95% CI
Calabresi 2014	1	358	3	355	7.6%	0.33 [0.03, 3.16]
Cree 2018	6	433	11	428	39.8%	0.54 [0.20, 1.44]
Cree 2020	4	345	7	324	26.0%	0.54 [0.16, 1.82]
Fox 2014	5	783	2	245	14.5%	0.78 [0.15, 4.01]
Kappos 2010	4	425	1	418	8.1%	3.93 [0.44, 35.05]
NCT01317004	1	50	0	11	3.9%	0.71 [0.03, 16.28]
**Total (95% CI)**		**2394**		**1781**	**100.0%**	**0.65 [0.35, 1.21]**
Total events	21		24			

Heterogeneity: Tau² = 0.00; Chi² = 3.25, df = 5 (P = 0.66); I² = 0%
Test for overall effect: Z = 1.36 (P = 0.17)

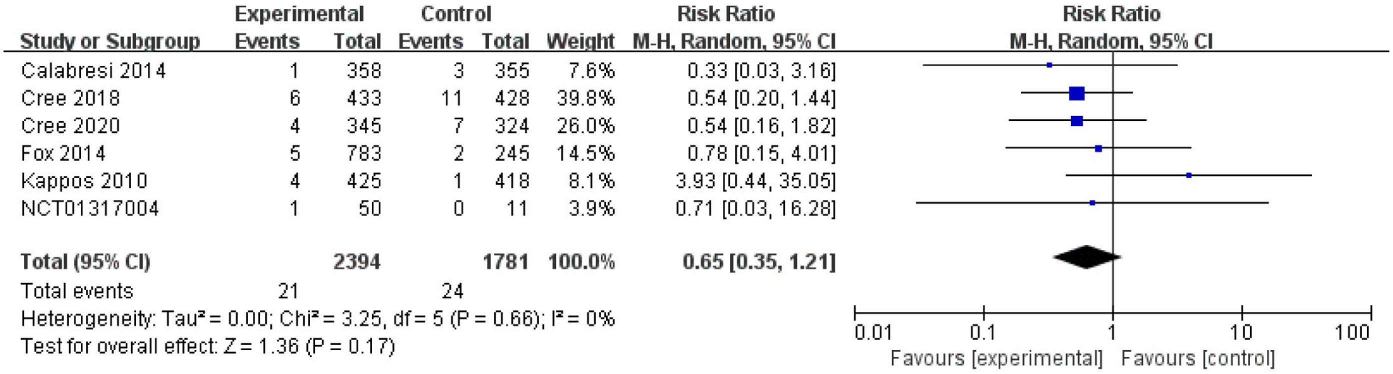

B. Death

Study or Subgroup	Experimental Events	Experimental Total	Control Events	Control Total	Weight	Risk Ratio M-H, Random, 95% CI	Year
Cohen 2010	0	429	0	431		Not estimable	2010
Kappos 2010	0	425	2	418	100.0%	0.20 [0.01, 4.09]	2010
Calabresi 2014	0	358	0	355		Not estimable	2014
Fox 2014	0	783	0	245		Not estimable	2014
**Total (95% CI)**		**1995**		**1449**	**100.0%**	**0.20 [0.01, 4.09]**	
Total events	0		2				

Heterogeneity: Not applicable
Test for overall effect: Z = 1.05 (P = 0.29)

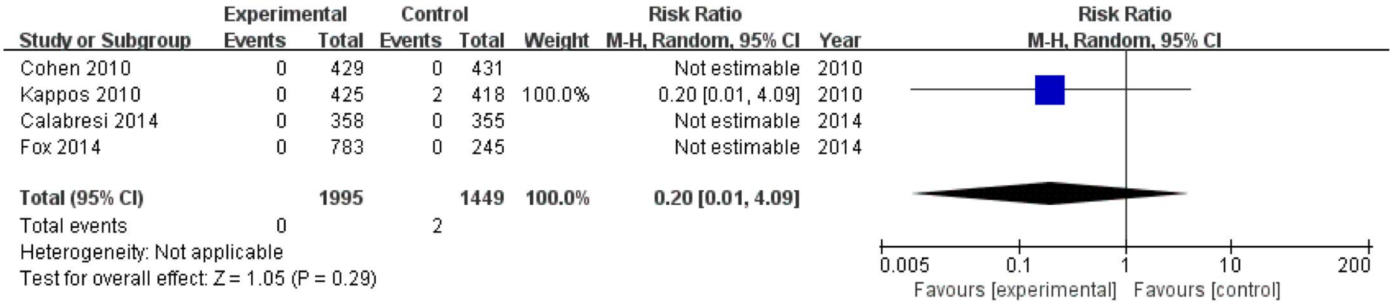

C. Lymphopenia

Study or Subgroup	Experimental Events	Experimental Total	Control Events	Control Total	Weight	Risk Ratio M-H, Random, 95% CI	Year
Kappos 2010	0	425	0	418		Not estimable	2010
Fox 2014	2	783	0	245	49.0%	1.57 [0.08, 32.57]	2014
NCT01317004	2	50	0	11	51.0%	1.18 [0.06, 22.94]	2015
**Total (95% CI)**		**1258**		**674**	**100.0%**	**1.35 [0.16, 11.31]**	
Total events	4		0				

Heterogeneity: Tau² = 0.00; Chi² = 0.02, df = 1 (P = 0.89); I² = 0%
Test for overall effect: Z = 0.28 (P = 0.78)

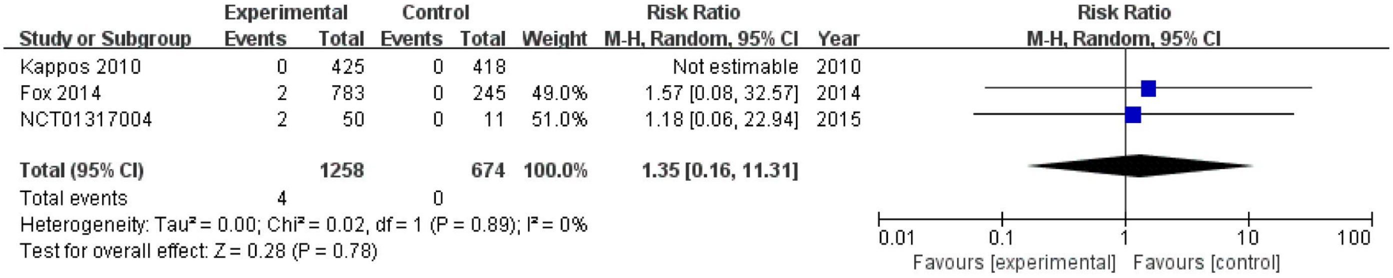

D. Epilepsy

Study or Subgroup	Experimental Events	Experimental Total	Control Events	Control Total	Weight	Risk Ratio M-H, Random, 95% CI	Year
Kappos 2010	0	425	0	418		Not estimable	2010
Calabresi 2014	3	358	1	355	66.7%	2.97 [0.31, 28.46]	2014
Cree 2020	1	345	0	324	33.3%	2.82 [0.12, 68.93]	2020
**Total (95% CI)**		**1128**		**1097**	**100.0%**	**2.92 [0.46, 18.48]**	
Total events	4		1				

Heterogeneity: Tau² = 0.00; Chi² = 0.00, df = 1 (P = 0.98); I² = 0%
Test for overall effect: Z = 1.14 (P = 0.25)

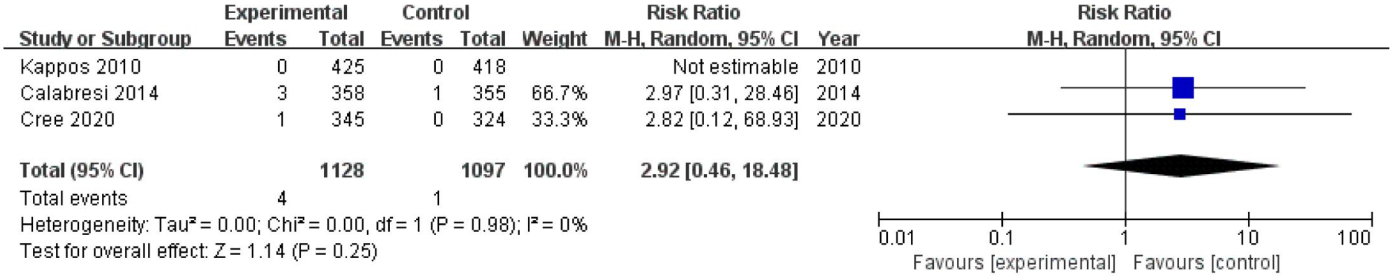

E. Depression

Study or Subgroup	Experimental Events	Experimental Total	Control Events	Control Total	Weight	Risk Ratio M-H, Random, 95% CI	Year
Kappos 2010	0	425	1	418	27.9%	0.33 [0.01, 8.03]	2010
Calabresi 2014	1	358	5	355	41.6%	0.20 [0.02, 1.69]	2014
Cree 2020	3	433	0	428	30.5%	6.92 [0.36, 133.55]	2020
**Total (95% CI)**		**1216**		**1201**	**100.0%**	**0.67 [0.08, 6.03]**	
Total events	4		6				

Heterogeneity: Tau² = 1.81; Chi² = 3.86, df = 2 (P = 0.15); I² = 48%
Test for overall effect: Z = 0.35 (P = 0.72)

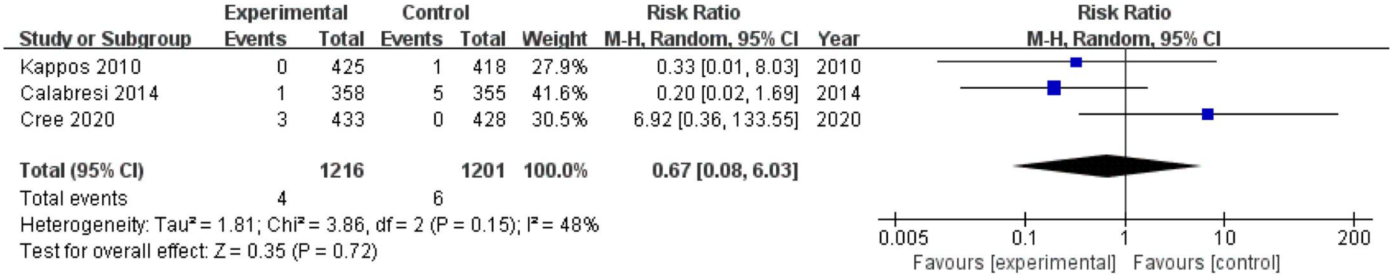

Fig. S13 (Sub-analysis of specific SAEs in the fingolimod 0.5 mg/d group)

A. Breast cancer

Study or Subgroup	Experimental Events	Experimental Total	Control Events	Control Total	Weight	Risk Ratio M-H, Random, 95% CI
Cohen 2010	2	429	0	431	49.6%	5.02 [0.24, 104.32]
Kappos 2010	0	425	3	418	50.4%	0.14 [0.01, 2.71]
**Total (95% CI)**		**854**		**849**	**100.0%**	**0.83 [0.02, 27.60]**
Total events	2		3			

Heterogeneity: Tau² = 4.07; Chi² = 2.74, df = 1 (P = 0.10); I² = 64%
Test for overall effect: Z = 0.11 (P = 0.92)

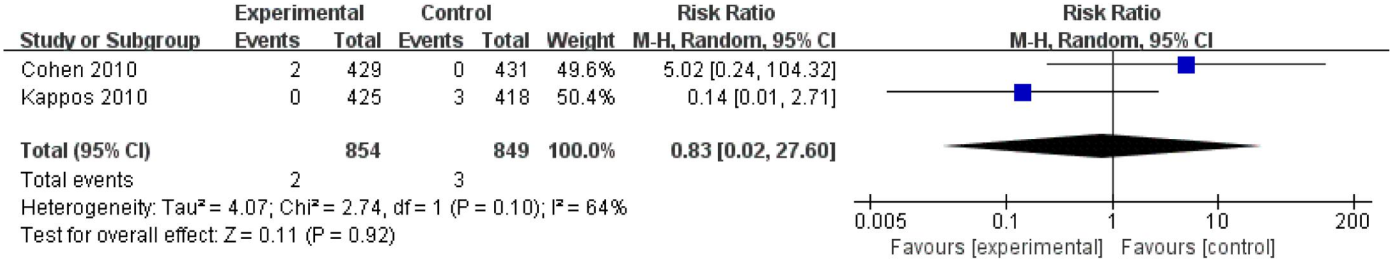

B. Melanoma

Study or Subgroup	Experimental Events	Experimental Total	Control Events	Control Total	Weight	Risk Ratio M-H, Random, 95% CI
Cohen 2010	0	429	0	431		Not estimable
Kappos 2010	0	425	1	418	100.0%	0.33 [0.01, 8.03]
**Total (95% CI)**		**854**		**849**	**100.0%**	**0.33 [0.01, 8.03]**
Total events	0		1			

Heterogeneity: Not applicable
Test for overall effect: Z = 0.68 (P = 0.49)

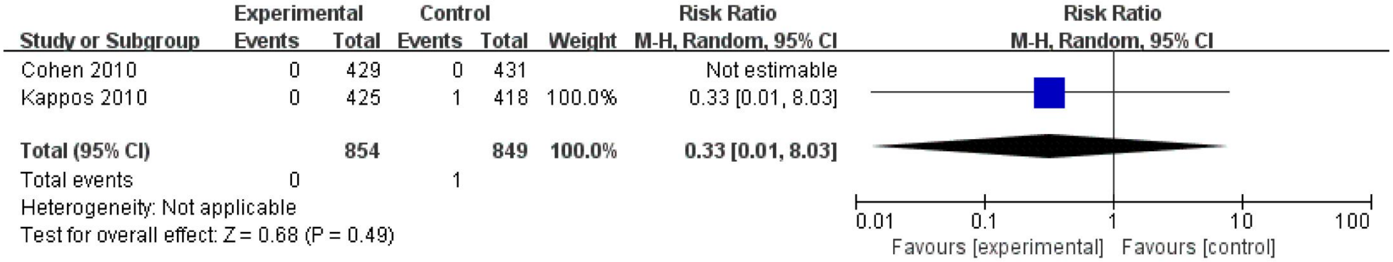

C. Abortion

Study or Subgroup	Experimental Events	Experimental Total	Control Events	Control Total	Weight	Risk Ratio M-H, Random, 95% CI	Year
Kappos 2010	0	425	3	418	39.6%	0.14 [0.01, 2.71]	2010
Calabresi 2014	1	358	2	355	60.4%	0.50 [0.05, 5.44]	2014
**Total (95% CI)**		**783**		**773**	**100.0%**	**0.30 [0.05, 1.94]**	
Total events	1		5				

Heterogeneity: Tau² = 0.00; Chi² = 0.43, df = 1 (P = 0.51); I² = 0%
Test for overall effect: Z = 1.26 (P = 0.21)

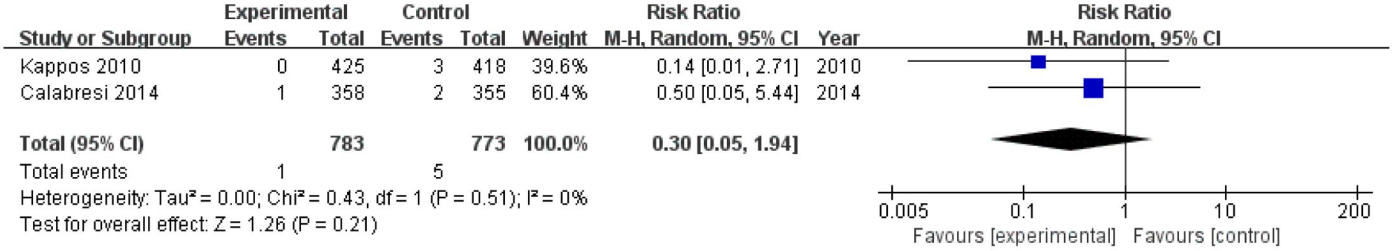

D. Syncope

Study or Subgroup	Experimental Events	Experimental Total	Control Events	Control Total	Weight	Risk Ratio M-H, Random, 95% CI
Calabresi 2014	2	358	1	355	58.0%	1.98 [0.18, 21.77]
Cree 2020	0	345	2	324	42.0%	0.19 [0.01, 3.90]
**Total (95% CI)**		**703**		**679**	**100.0%**	**0.74 [0.07, 7.34]**
Total events	2		3			

Heterogeneity: Tau² = 0.88; Chi² = 1.45, df = 1 (P = 0.23); I² = 31%
Test for overall effect: Z = 0.26 (P = 0.79)

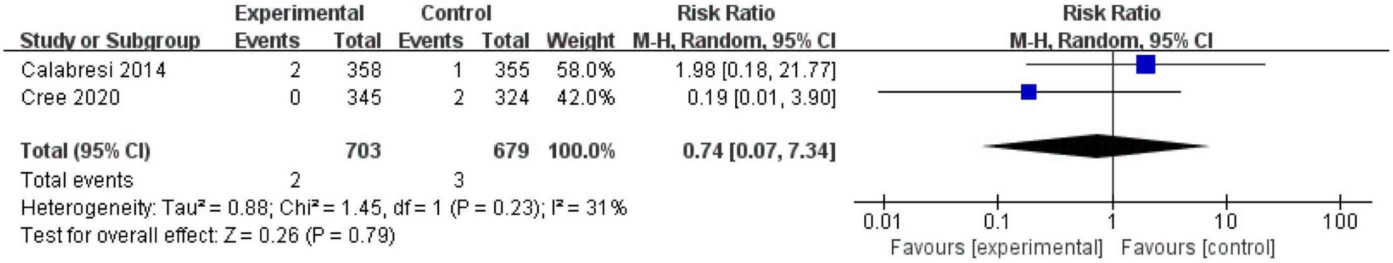

E. Macular edema

Study or Subgroup	Experimental Events	Experimental Total	Control Events	Control Total	Weight	Risk Ratio M-H, Random, 95% CI	Year
Kappos 2010	0	425	0	418		Not estimable	2010
Calabresi 2014	1	358	1	355	100.0%	0.99 [0.06, 15.79]	2014
**Total (95% CI)**		**783**		**773**	**100.0%**	**0.99 [0.06, 15.79]**	
Total events	1		1				

Heterogeneity: Not applicable
Test for overall effect: Z = 0.01 (P = 1.00)

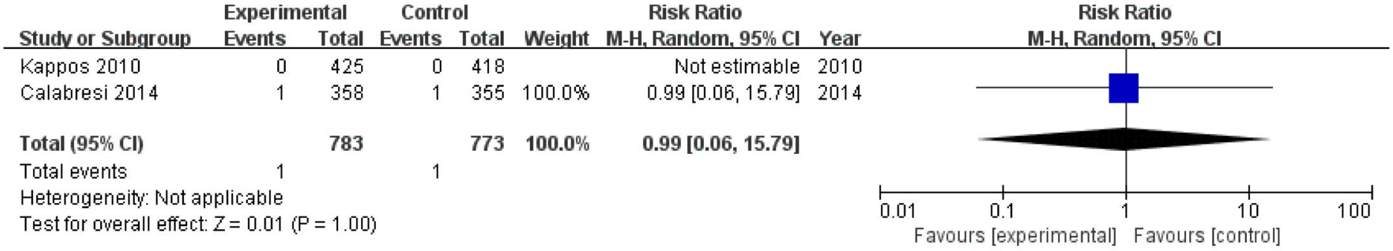

Fig. S14 (Sub-analysis of basal-cell carcinoma in the fingolimod 0.5 mg/d group)

	Experimental		Control			Risk Ratio	
Study or Subgroup	Events	Total	Events	Total	Weight	M-H, Random, 95% CI	Year
Cohen 2010	3	429	1	431	20.5%	3.01 [0.31, 28.86]	2010
Kappos 2010	4	425	1	418	21.8%	3.93 [0.44, 35.05]	2010
Calabresi 2014	10	358	2	355	45.8%	4.96 [1.09, 22.47]	2014
Cree 2020	3	345	0	324	11.9%	6.58 [0.34, 126.80]	2020
**Total (95% CI)**		**1557**		**1528**	**100.0%**	**4.40 [1.58, 12.24]**	
Total events	20		4				

Heterogeneity: Tau² = 0.00; Chi² = 0.21, df = 3 (P = 0.98); I² = 0%

Test for overall effect: Z = 2.84 (P = 0.004)

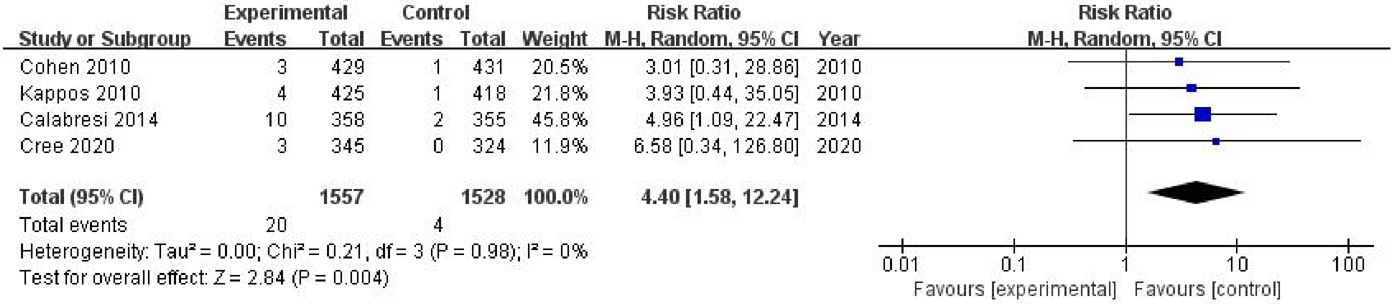

Fig. S15

A

Study or Subgroup	Experimental Mean	Experimental SD	Experimental Total	Control Mean	Control SD	Control Total	Weight	Mean Difference IV, Random, 95% CI	Year
**1.1.1 fingolimod 0.25mg**									
Cree 2020	0.22	0.49	366	0.26	0.55	324	100.0%	-0.04 [-0.12, 0.04]	2020
**Subtotal (95% CI)**			**366**			**324**	**100.0%**	**-0.04 [-0.12, 0.04]**	
Heterogeneity: Not applicable									
Test for overall effect: Z = 1.00 (P = 0.32)									
**1.1.2 fingolimod 0.5mg**									
Cohen 2010	0.16	0.42	429	0.33	0.74	431	18.9%	-0.17 [-0.25, -0.09]	2010
Kappos 2010	0.18	0.32	425	0.4	0.63	418	21.8%	-0.22 [-0.29, -0.15]	2010
Saida 2012	0.5	0.81	57	0.99	1.23	57	1.8%	-0.49 [-0.87, -0.11]	2012
Calabresi 2014	0.21	0.39	358	0.4	0.58	355	20.6%	-0.19 [-0.26, -0.12]	2014
Cree 2018	0.22	0.53	433	0.31	0.84	428	16.2%	-0.09 [-0.18, 0.00]	2018
Cree 2020	0.15	0.38	345	0.26	0.55	324	20.7%	-0.11 [-0.18, -0.04]	2020
**Subtotal (95% CI)**			**2047**			**2013**	**100.0%**	**-0.17 [-0.22, -0.11]**	
Heterogeneity: Tau² = 0.00; Chi² = 10.47, df = 5 (P = 0.06); I² = 52%									
Test for overall effect: Z = 6.22 (P < 0.00001)									
**1.1.3 fingolimod 1.25mg**									
Kappos 2010	0.16	0.32	429	0.4	0.63	418	33.1%	-0.24 [-0.31, -0.17]	2010
Cohen 2010	0.2	0.42	420	0.33	0.74	431	29.8%	-0.13 [-0.21, -0.05]	2010
Saida 2012	0.41	0.71	54	0.99	1.23	57	3.9%	-0.58 [-0.95, -0.21]	2012
Calabresi 2014	0.2	0.29	370	0.4	0.58	355	33.2%	-0.20 [-0.27, -0.13]	2014
**Subtotal (95% CI)**			**1273**			**1261**	**100.0%**	**-0.21 [-0.28, -0.13]**	
Heterogeneity: Tau² = 0.00; Chi² = 8.27, df = 3 (P = 0.04); I² = 64%									
Test for overall effect: Z = 5.30 (P < 0.00001)									

Test for subgroup differences: Chi² = 10.04, df = 2 (P = 0.007), I² = 80.1%

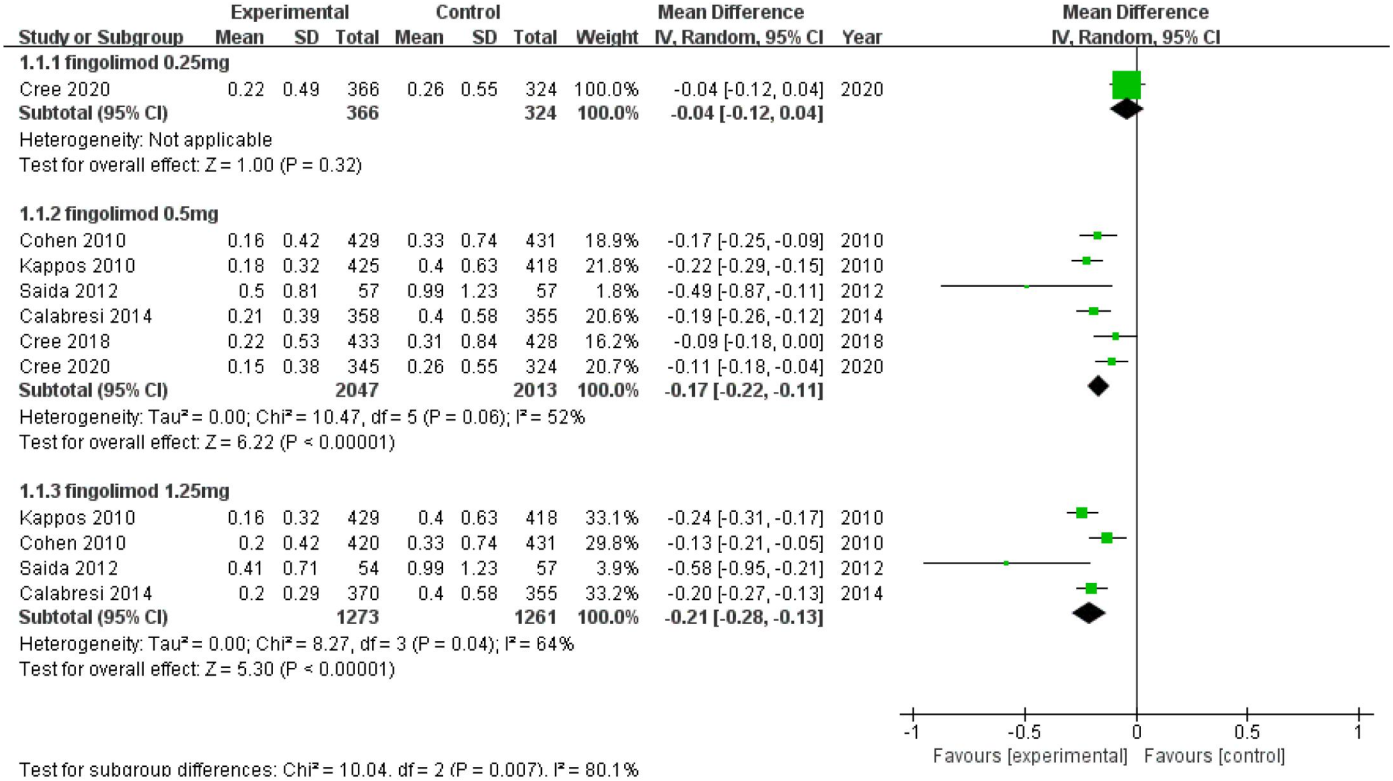

B

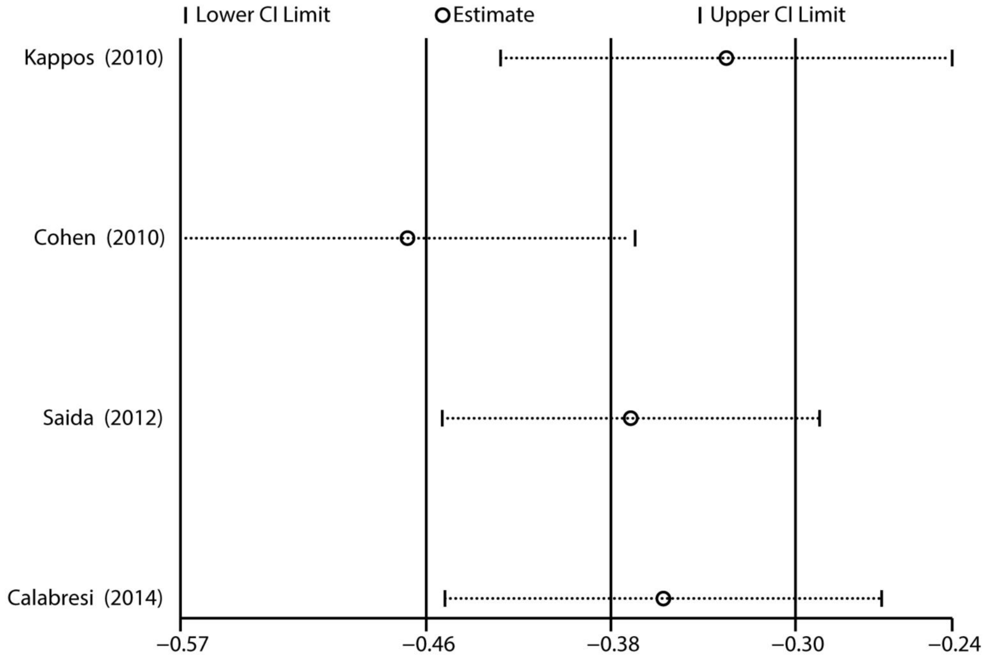

Fig. S16

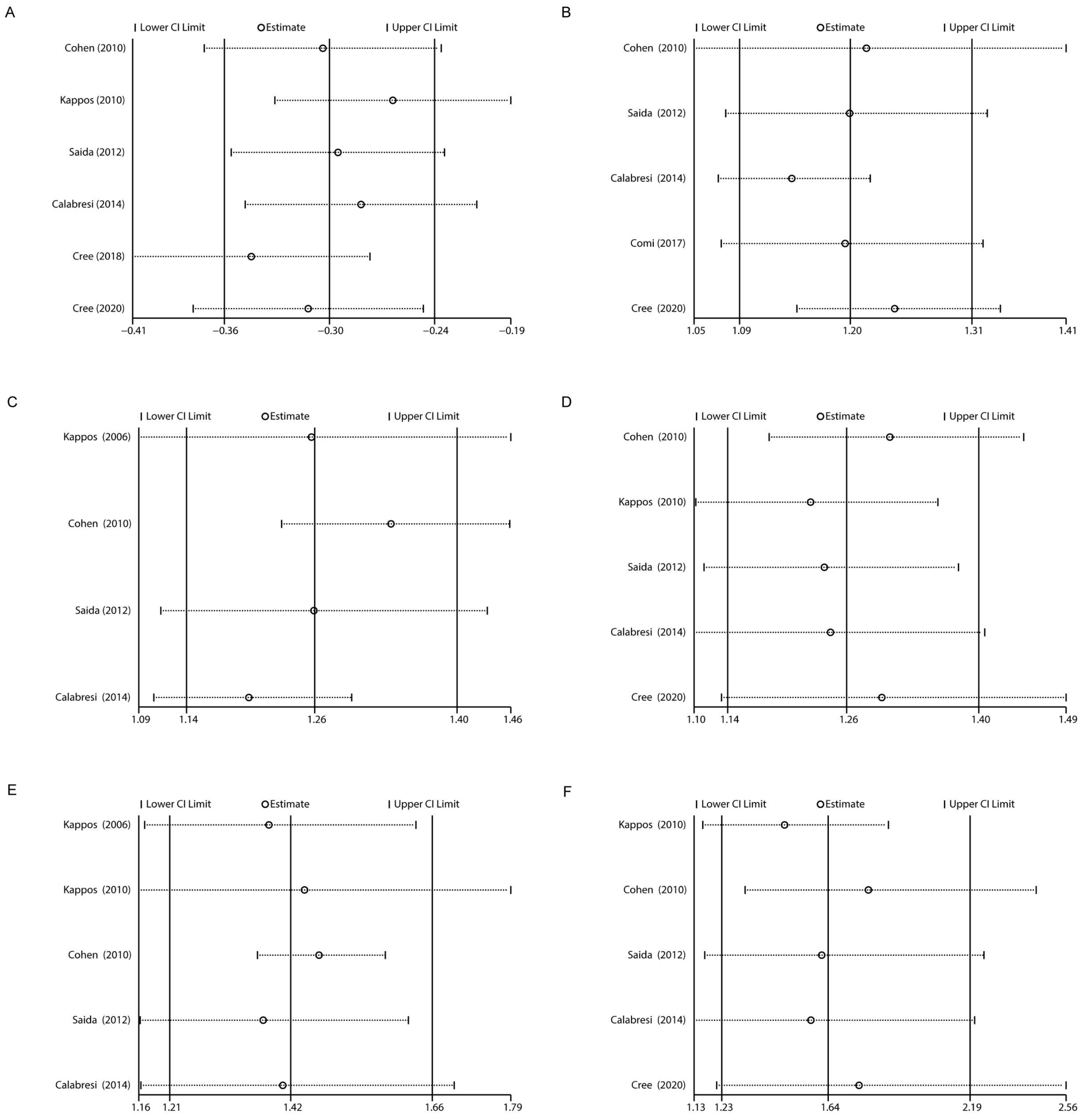
A
Lower CI Limit
Estimate
Upper CI Limit
Cohen (2010)
Kappos (2010)
Saida (2012)
Calabresi (2014)
Cree (2018)
Cree (2020)
−0.41
−0.36
−0.30
−0.24
−0.19
B
Lower CI Limit
Estimate
Upper CI Limit
Cohen (2010)
Saida (2012)
Calabresi (2014)
Comi (2017)
Cree (2020)
1.05
1.09
1.20
1.31
1.41
C
Lower CI Limit
Estimate
Upper CI Limit
Kappos (2006)
Cohen (2010)
Saida (2012)
Calabresi (2014)
1.09
1.14
1.26
1.40
1.46
D
Lower CI Limit
Estimate
Upper CI Limit
Cohen (2010)
Kappos (2010)
Saida (2012)
Calabresi (2014)
Cree (2020)
1.10
1.14
1.26
1.40
1.49
E
Lower CI Limit
Estimate
Upper CI Limit
Kappos (2006)
Kappos (2010)
Cohen (2010)
Saida (2012)
Calabresi (2014)
1.16
1.21
1.42
1.66
1.79
F
Lower CI Limit
Estimate
Upper CI Limit
Kappos (2010)
Cohen (2010)
Saida (2012)
Calabresi (2014)
Cree (2020)
1.13
1.23
1.64
2.19
2.56

Fig. S17

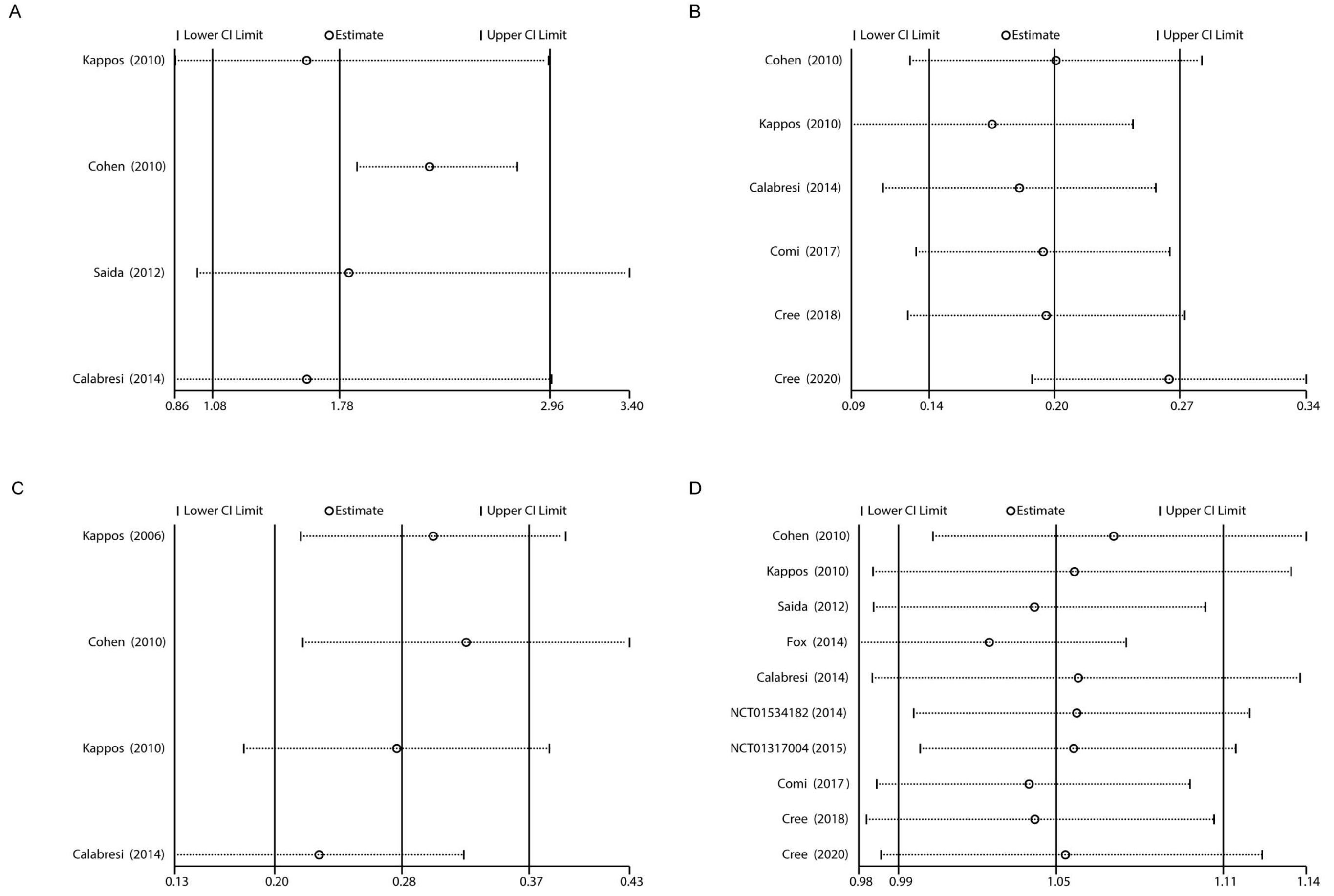
A
Lower CI Limit
Estimate
Upper CI Limit
Kappos (2010)
Cohen (2010)
Saida (2012)
Calabresi (2014)
0.86
1.08
1.78
2.96
3.40
B
Lower CI Limit
Estimate
Upper CI Limit
Cohen (2010)
Kappos (2010)
Calabresi (2014)
Comi (2017)
Cree (2018)
Cree (2020)
0.09
0.14
0.20
0.27
0.34
C
Lower CI Limit
Estimate
Upper CI Limit
Kappos (2006)
Cohen (2010)
Kappos (2010)
Calabresi (2014)
0.13
0.20
0.28
0.37
0.43
D
Lower CI Limit
Estimate
Upper CI Limit
Cohen (2010)
Kappos (2010)
Saida (2012)
Fox (2014)
Calabresi (2014)
NCT01534182 (2014)
NCT01317004 (2015)
Comi (2017)
Cree (2018)
Cree (2020)
0.98
0.99
1.05
1.11
1.14
